# Structural Characterization and Multiomics Analysis Reveal Extensive Diversity and Global Distribution of Kurstakin Lipopeptides

**DOI:** 10.1021/acs.jnatprod.5c01212

**Published:** 2025-11-27

**Authors:** Rose Campbell, Emily Mevers

**Affiliations:** Department of Chemistry, 1757 Virginia Tech, Blacksburg, Virginia 24060, United States

## Abstract

*Bacillus* species, particularly those investigated as biocontrol agents, are known to produce a cocktail of bioactive lipopeptides that act synergistically to shape the ecological function of these beneficial microbes. However, while certain families of lipopeptides are well-characterized, others remain elusive. Herein, we describe the characterization of the kurstakins, a family of lipopeptides associated with promising biocontrol properties but that lack adequate characterization. Metabolomic analyses of a semipurified *Bacillus cereus* EM195W extract fraction revealed the presence of approximately 50 cyclic- and linear-peptide analogs. Deeper analyses revealed that the chemical diversity stems from the diverse lipid tails, including linear, *iso*-, and *anteiso*-lipid tails ranging from C_8_–C_18_, along with several hydroxylated lipid tails. Isolation and complete structural analysis of two new analogs represented the first kurstakin analogs characterized by NMR and provided the first experimental analyses for deducing their absolute configuration. Finally, analysis of publicly available genomic and MS data provided insights into the true chemical diversity and distribution of the kurstakins. These results expand our understanding of this family of compounds, opening the door for determining their ecological functions and the role they play in the broader activity of biocontrol agents.

## Graphical abstract

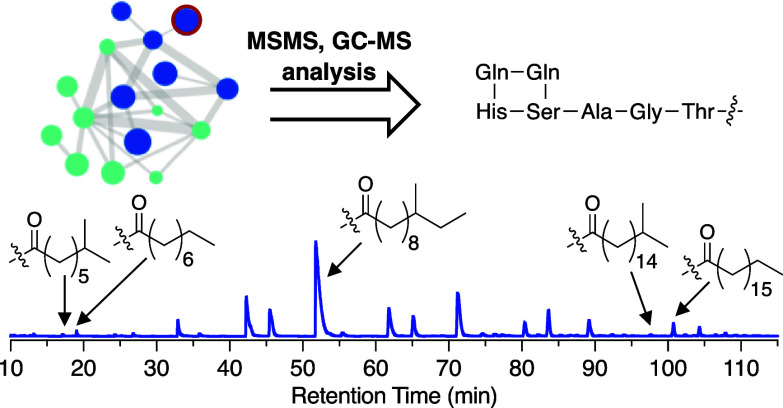

Lipopeptides are a well-known family of microbial natural products that possess a range of applications, including their potential medicinal uses and as crop biocontrol agents.^1^ Several lipopeptides or synthetic analogs are FDA-approved for the treatment of infectious diseases, including as antibiotics (*e.g.,* polymyxin B, oritavancin, telavancin, and daptomycin) and antifungals (*e.g.,* rezafungin and micafungin), with many others in various stages of clinical trials.^2,3^ Their bioactive properties arise from their amphiphilic structures, which consist of a fatty acid tail (lipid) and a polar peptide. The lipopeptide antibiotics are known to bind to cell membranes, forming pores that lead to depolarization.^4^ Lipopeptides are commonly produced by a diverse range of microorganisms, including bacteria, fungi, and cyanobacteria; however, Bacilli are particularly well-known for their ability to produce structurally diverse bioactive lipopeptides. This includes the widespread production of surfactins, iturins, fengycins, polypeptins, and octapeptins.^2,5^ This biosynthetic capability has led to their wide use as biocontrol agents in agricultural settings, such as *Bacillus thuringiensis* being leveraged for insect and disease control and *Bacillus velezensis* for controlling plant pathogens.^6,7^ The diverse lipopeptides produced by these strains have been shown to work synergistically as antifungals, biofilm formation regulators, and plant defense modulators.^5,8^

Although many lipopeptides have been well characterized from Bacilli, this does not represent their full biosynthetic potential.^9^ In addition, many lipopeptide families have been mentioned in the literature, but lack appropriate analytical data to confirm their planar and secondary structures and the diversity of the family.^2^ This includes the kurstakins, gavaserin, and saltavalin, which have all been implicated to play a critical role in the biocontrol properties of the producing organisms.^10^ The kurstakins were first discovered to be produced by *B. thuringiensis*, and have been considered a biomarker for *Bacillus cereus* group species.^11,12^ However, they have since been isolated from a range of bacteria, including non-*cereus* group *Bacillus* species (*e.g., B*. *subtilis*, *B*. *licheniformis*)^13,14^ and more distantly related bacteria (*e.g., Enterobacter cloacae*, and *Citrobacter* sp.).^15,16^ Several of the producing strains have been studied extensively for their potential use as a biocontrol agent, due to their antimicrobial activity and ability to swarm and produce protective biofilms,^12,17,18^ which has been attributed in part to the kurstakins.^19–22^ Four cyclic analogs, kurstakins 1–4 (**1**–**4**), were elucidated through tandem mass spectrometry (MSMS) analyses and chemical derivatization.^12^ There have been reports of many additional analogs, but these reports are primarily based on MS analyses.^23–25^ The studies indicate that all analogs contain the same peptide core, but incorporate diverse lipid tails. Additionally, amino acid configurations were deduced through bioinformatic analyses alone,^17^ which can only predict the configuration of the α-position. Therefore, chemical analyses are needed to unequivocally determine the amino acid configurations, particularly of the β-hydroxyl in the threonine residue. Herein, we leveraged our metabolomic data set from marine bacteria to characterize the kurstakins, including the first NMR analysis, report of two new analogs, kurstakins 5 and 6 (**5**–**6**), deduction of the absolute configuration, and analysis of the true chemical diversity and distribution of the kurstakins.
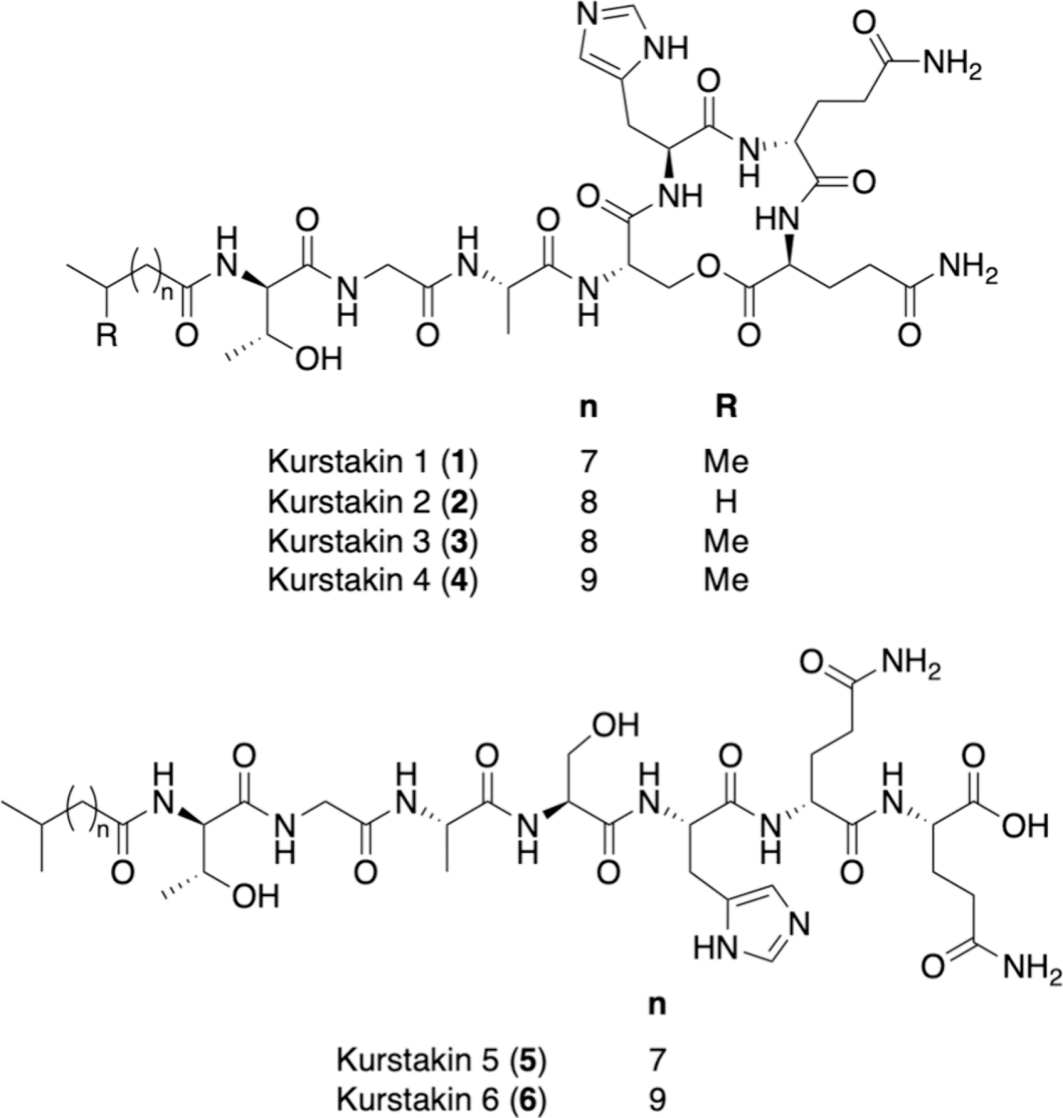


## Results and Discussion

Analysis of a Global Natural Product Social (GNPS) molecular network representing the chemical potential of diverse marine bacteria from our internal library revealed that it contained a rather large spectral family, with masses ranging from 850 to 940 *m*/*z* (Figure 1A).^26^ A subset of these nodes were identified as **1**–**4** based on their exact mass and key fragment ions (Figure 1B). A peak at 110.1 *m*/*z*, which putatively represents a decarboxylated histidine, was abundant in all MSMS spectra and became a key identifying fragment (). Other nodes matched masses of reported kurstakin analogs with hypothetical structures,^15,19,23,27^ as well as new analogs. Based on key MS fragments, this spectral family represents both cyclic and linear core peptide sequences that have incorporated various lipid tails (*e.g.,* C_9_–C_14_ with and without hydroxylation; ).

**Figure 1:**
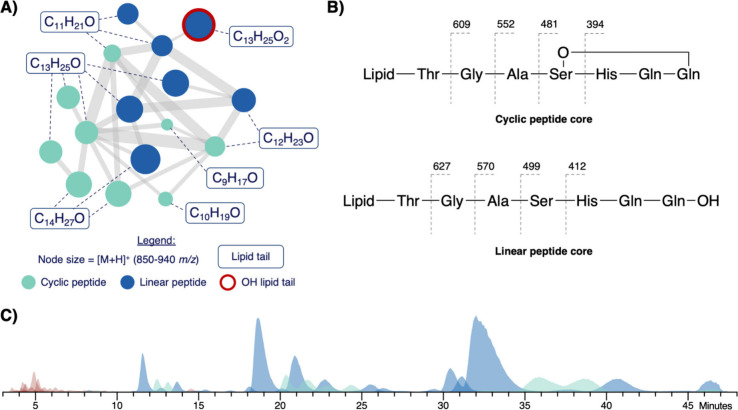
(A) Molecular family of kurstakins found in the marine isolates library, showing structural diversity in tail structure and peptide linearity. (B) Key common fragments of each core peptide used to identify the kurstakins. Fragments not including histidine were rarely observed. (C) Overlaid EICs of all kurstakin masses observed in an enriched semipure fraction, 850–996 *m*/*z*, representing a large diversity of cyclic (aqua), linear (blue), and hydroxyl-tail (red) analogs.

To characterize the structural breadth of the kurstakin family, the producing strain, *B. cereus* EM195W, was grown in large-scale (8 L) liquid media containing hydrophobic resins (HP-20, XAD4, and XAD7) to capture the secreted small molecules. After 9 days, the resin was extracted with organic solvents to generate a crude extract. The crude extract was first subjected to reverse-phase (RP) flash chromatography to yield eight refined fractions. MS analysis revealed that the kurstakins were present in the nonpolar fractions, which were combined and processed through normal-phase (NP) flash chromatography, generating an additional 13 fractions. Fractions that were highly enriched with the kurstakins were reanalyzed by high-resolution liquid chromatography-MS (HR-LCMS), revealing the presence of many more analogs than previously detected in the original GNPS analysis. Manual interpretation of the MS data indicated that there is significantly more tail variation than previously thought, which was likely overlooked due to the isobaric nature of many of the analogs and low relative abundance of several analogs (Figure 1C). In total, 31 masses ranging from 850–996 *m*/*z* were detected, representing approximately 50 distinct analogs (). The breadth of these analogs spanned tail lengths ranging from C_9_–C_17_, most of which appeared as all possible combinations of linear- or cyclic-peptide core with and without hydroxylated tails, and represented many previously undescribed compounds.

To discover the breadth of tail diversity, we turned to gas chromatography-MS (GC-MS) analysis. A portion of one of the semipurified NP fractions was treated with HCl (*aq*) to liberate the free acid, which was subsequently methylated with TMS-diazomethane. The methylated fatty acids were analyzed by GC-MS, comparing the retention times and fragmentation patterns with authentic standards of linear, *iso*-branched, and *anteiso*-branched fatty acid methyl esters (LCFA, *i*BCFA, and *a*BCFA, respectively).^34^ Comparing the derivatized natural material to the LCFA standards revealed the presence of linear fatty acid tails ranging in length from C_9_–C_18_ (Figure 2A). This agreed with the masses found on the HR-LCMS, but with the addition of the C_18_ LCFA.

**Figure 2:**
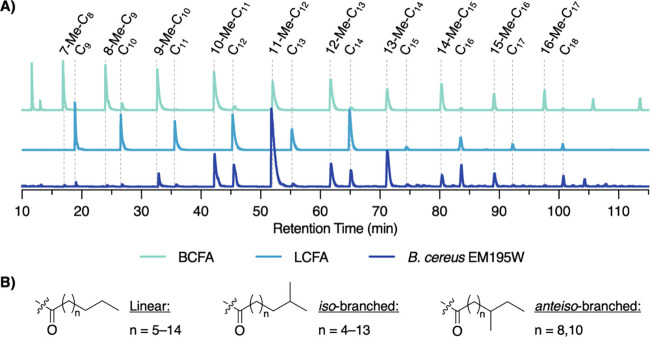
Chemical characterization of the family of kurstakins. (A) GC-MS traces (EIC of common 74.1 *m*/*z* fragment) of the semipure *B. cereus* EM195W fraction (purple) compared to the LCFA (blue) and *i*BCFA (aqua) standards. (B) Summary of all fatty acid tails identified in kurstakins produced by EM195W.

Both the *i*BCFA and *a*BCFAs standards coeluted after their corresponding LCFA of the same length, so they were indistinguishable from one another by retention time alone (). Analysis of the derivatized natural product mixture indicated the presence of branched lipids ranging from Me-C_8_ to Me-C_17_ (Figure 2A). To identify these as *i*BCFA or *a*BCFA, we used a key fragment ion;^12^ all *a*BCFA standards contain a diagnostic neutral loss of 61 Da—hypothesized to result from the loss of the *O*-methyl and terminal C_2_H_5_—which is absent in the *i*BCFA standards (). Searching the BCFA peaks of the derivatized natural material for the presence of this diagnostic neutral loss revealed the presence of two *a*BCFA tails, 10-Me-C_12_ and 12-Me-C_14_ (). In the spectra of both these peaks, the relative abundance of the peak representing this neutral loss was significantly lower (0.49 and 0.75%, respectively) than that of the pure *a*BCFA standards (3.02 and 1.85%, respectively), suggesting that these peaks contained a mixture of both *a*BCFA and *i*BCFA isomers. All other BCFA peaks detected were thus attributed to *i*BCFA, indicating the presence of lipids ranging from 7-Me-C_8_ to 16-Me-C_17_, which closely matches the LCFA data. In total, twenty-two distinct lipid tails were detected (Figure 2B). Notably, BCFAs were consistently 2–13-fold more abundant than their LCFAs analogs for chain lengths C_10–15_, suggesting there may be slight branched-chain lipid selectivity in kurstakin biosynthesis.

HPLC purification of fractions from the large-scale extract resulted in the isolation of two major linear-peptide kurstakin analogs, 9-Me-C_10_-kurstakin (**5**) and 11-Me-C_12_-kurstakin (**6**), which were fully characterized through traditional NMR techniques (^1^H, gHSQC, TOCSY, H2BC, and HMBC). Initial attempts at acquiring NMR data in *d*_6_-DMSO led to missing correlations, particularly within the basic functionalities of the histidine and glutamine residues, and overall, the ^1^H NMR spectra displayed significant line broadening. To improve the spectra quality, trifluoroacetic acid (TFA) fumes were bubbled into the NMR sample until both the exchangeable protons and aromatic histidine protons (δ_H_ 7.36 and 8.93) sharpened, and the residual water peak shifted to approximately 5 ppm in the ^1^H NMR spectrum (). Detailed analysis of the 2D NMR data acquired on the acidified samples led to complete assignments of the ^1^H and ^13^C NMR chemical shifts, thereby confirming the amino acid sequence as previously reported ().^12^ In addition, **5** and **6** contain 9-Me-C_10_ and 11-Me-C_12_ lipids, respectively, which represent the most abundant lipids from the GC-MS analysis.

Marfey’s analysis of **5** and of an enriched NP fraction was used to unequivocally assign the configuration of the peptide core (). Previous studies identified epimerization domains in modules one and six of the biosynthetic gene cluster (BGC), corresponding to the threonine and the glutamine in the penultimate position,^17^ which led to their proposed assignment of d-configuration. However, the configuration of the β-hydroxyl of threonine has remained undefined. To assign the threonine configuration and confirm the configuration of the remaining residues, a small aliquot of both the semicrude fraction and purified **5** was hydrolyzed, then reacted with l-FDAA. The derivatized products were analyzed by low-resolution (LR)-LCMS and compared to authentic standards. This analysis revealed the presence of l-Ser (37.1 min), d-*allo*-Thr (45.5 min), l-Ala (57.0 min), l-His (69.9 min), and both l- and d-Gln (48.2 and 53.0 min, respectively) in both samples, indicating the kurstakins all contain the same heptapeptide and identifying Thr as d-*allo*-Thr, contrasting with the previous assumption that the kurstakins contain d-Thr.^11^ To unequivocally assign the stereoconfiguration of the two Gln residues and compare the BGC of *B. cereus* EM195W to the previously identified kurstakin BGC, we performed a genomic analysis. *B. cereus* EM195W genomic DNA was sequenced using Illumina, assembled by SeqCenter, and identified by the Type Genome Server.^28^ AntiSMASH 8.0 analysis of both the assembled genome and that of the known producer, *B. thuringiensis* 4BA1, identified the kurstakin BGC (*krs*) with seven adenylation domains predicted to load the amino acids observed in kurstakins.^17,29^ BLAST comparison of these two identified BGC sequences showed 96% similarity, indicating high similarity between biosynthesis of the kurstakins by *B. cereus* EM195W and the previously studied producer. Detailed analysis of the BGC revealed the presence of two epimerization domains in the same modules, one and six (), thereby confirming the observed d- and l-Gln are the sixth and seventh residues, respectively.

The kurstakins have been widely studied for their antimicrobial properties and the potential of the producing strain to be used as a biocontrol agent in agriculture; however, there is a lack of understanding of the global distribution of these important lipopeptides.^11^ Previously, the kurstakins have been thought to be biomarkers of *B. cereus* group species, yet it has been reported that only 15% (out of 123 genomes) of *Bacillus* strains contain the BGC.^9^ To gain an understanding of the phylogenetic diversity of bacterial producers, we examined the distribution of the BGC within publicly available genomes. First, *krs*A-C genes were translated, and the resulting protein sequence was analyzed in BLASTp. This resulted in the identification of over 3,000 NCBI proteins containing the *krs* BGC (See ). The phylogenetic tree of producing strains revealed that there are many potential producers of the kurstakins, with the vast majority within *B. cereus* group species (Figure 3A). Interestingly, several non-*Bacillus* strains also contain the BGC, including strains of *Priestia* spp., *Croceifilum oryzae*, and *Streptococcus pneumoniae*. To confirm that the quality control filters were not too loose, two representative non-*Bacillus* hits were analyzed by AntiSMASH to confirm the presence of the entire *krs* BGC. In addition, to mitigate alignment bias toward *Bacillus* sequences, the BLASTp analysis was performed again using a phylogenetically distant putative producer, *Croceifilum oryzae* DSM 46876, which resulted in a highly similar phylogenetic tree (). Although this broadly aligns with the previous literature that the kurstakins are predominantly produced by *B. cereus* group species, it is noteworthy that a subset of likely producers are from other diverse taxonomic groups.

**Figure 3:**
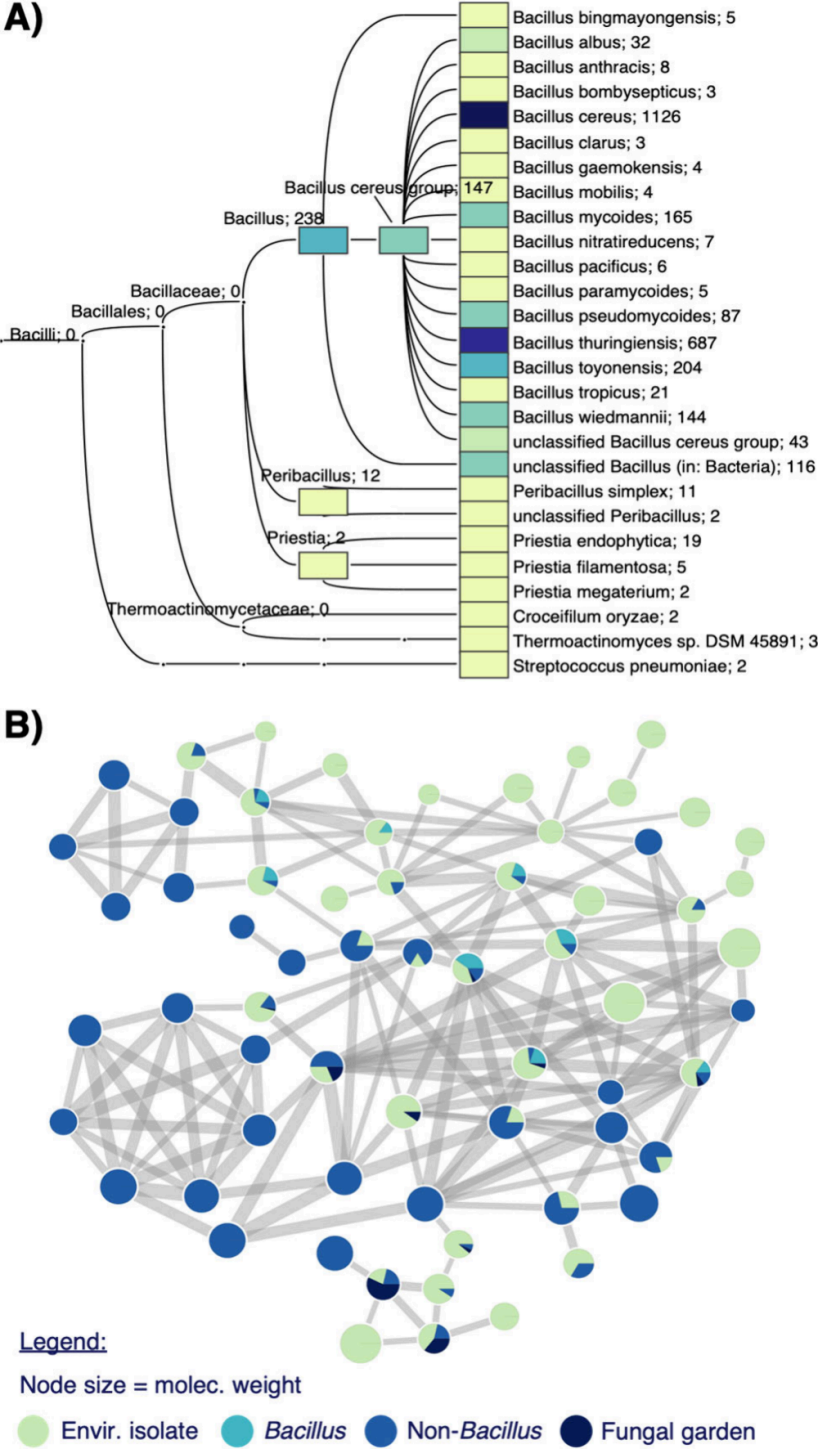
Leveraging public data to characterize kurstakin production. (A) Phylogenetic heatmap of NCBI strains identified to contain the *krs* gene cluster. (B) GNPS molecular family of kurstakins identified by propagated FASST searching, showing presence of kurstains in data sets from unidentified environmental isolates (green), identified *Bacillus* (teal) and non-*Bacillus* (blue) strains, and fungal garden extracts (navy).

Next, we leveraged public mass spectrometry data to determine where the kurstakins have been detected. Public data was queried by performing propagated FASST analysis using the GNPS2 platform to identify spectra related to the kurstakins in the public domain.^30^ This analysis uncovered over 300 instances of kurstakin analogs across 16 distinct massIVE (MSV) data sets, ranging from microbial monocultures to extracts of dynamic environmental communities, such as fungal gardens of ants (). Analysis of a molecular network of all samples identified to contain kurstakins revealed a wide range of kurstakin analogs, including masses of those identified in our work above and several new, unidentified masses (Figure 3B, ). These new analogs range from 850–998 *m*/*z*; however, based on MSMS fragments, the vast majority appear to contain the same heptapeptide but with further minor modifications. The nodes within this molecular family represent isolated strains from caves, plants, soil, and human skin, indicating that kurstakins are produced in diverse environmental niches. The results align with the above genomic analysis, considering that Bacilli are found in virtually all environmental niches probed by humans.^31^ Interestingly, kurstakins were detected in metabolomic data representing monocultures of Actinomycetes, α-proteobacteria (*Methylobacteria* spp.), and β-proteobacteria (*Burkholderia cenocepacia*), classes unrelated to *Bacillus*, which is dissimilar from the genomic analysis (). Although the cause of this disparity is unclear, we hypothesize that either this represents convergent evolution, isolated events of horizontal gene transfer in strains not available in the NCBI database, or contamination of the metabolomic data samples by a kurstakin-producing strain. However, we could not pursue this further as the strains identified by metabolomics are not commercially available and have no published genomic data.

In conclusion, through this study, we have illuminated the true chemical diversity of the kurstakins family of lipopeptides. Approximately 50 kurstakins were detected, with structural diversity arising mainly through structurally distinct lipid tails incorporated. Isolation of two new analogs led to the first NMR characterization of any member of the kurstakins and confirmation of the absolute configuration. Finally, a global analysis leveraging publicly available data led to the identification of numerous new potential producing strains and a deeper understanding of where these important compounds are produced. The combination of BLASTp and FASST analyses forms a complementary workflow for characterizing global production of kurstakins and identifying producers; while genomics led to hypothetical producers with no information on potential analogs produced, mass spectrometry results revealed observed production and structural insight. Conversely, public mass spectrometry data lacks evolutionary insight and is limited in quantity and standardization, which genomic analyses can provide. These insights will inform future work toward developing a kurstakin producer as a biocontrol agent.

## Experimental Section

### General Experimental Procedures

Optical rotation data were recorded on a JASCO P-2000 polarimeter. NMR spectra were recorded in *d*_6_-DMSO with the residual solvent peak as an internal standard (δ_C_ 39.5, δ_H_ 2.50) on a Bruker Avance III 600 MHz instrument equipped with a triple resonance inverse (CP-TCI) Prodigy N_2_ cooled CryoProbe (^1^H 600 MHz; ^13^C 150 MHz) or a Bruker Avance II 500 MHz instrument equipped with a CPBBO Prodigy N_2_ cooled CryoProbe (^1^H 500 MHz; ^13^C 125 MHz). TFA fuming was monitored on a JEOL ECZ 400 MHz instrument equipped with a Royal HFX probe (^1^H 400 MHz). GC-MS data were obtained using an Agilent 5975C VL MSD GC-MS. LR-LCMS data were obtained using an Agilent 1200 series HPLC system equipped with a photodiode array detector and a Thermo LTQ mass spectrometer. HR-ESIMS and HR-MSMS were carried out using an Agilent 6530 Q-TOF equipped with a 1290 Infinity II UPLC system or a Thermo Orbitrap Exploris 120 mass spectrometer. Reverse-phase flash chromatography purification was carried out with a Biotage Selekt automated flash chromatography system. HPLC purifications were carried out using a 1260 Infinity II HPLC system equipped with a photodiode array detector. All solvents were of HPLC quality.

### Collection Sites for Bacterial Isolation

Egg collars used to isolate bacterial strains were collected from three sites in SW Florida across multiple seasons—Dec. 2018, Dec. 2019, Feb. 2023, and Jan. 2024 (permit number: SAL-19-2113-SR; GPS coordinates: 26.591972, −82.068167; 26.681361, −82.089000; and 26.665222, −82.105278)—and one site in W. Puerto Rico in Dec. 2022 (GPS coordinates: 17.978961, −67.213472). All egg masses were found in 2–4 ft of water. On the same day as collection, small portions of each egg collar were surface sterilized by submerging in 70% ethanol (aq) for 30 s, rinsed three times with sterile water, and then ground in sterile water using a sterilized glass rod. Aliquots of this liquid were plated on various solid agar media for isolation, including marine agar, R2A in seawater, chitin in seawater, and RAM in seawater, all containing 50 mg/L of nystatin and cycloheximide.^32^ Plates were incubated at room temp (rt) and monitored for bacterial growth. Colonies were subinoculated onto yeast extract malt extract (YEME) and this process was repeated until pure cultures were obtained. All isolated bacterial strains were cryopreserved in 25% glycerol/water and identified to the genus level using 16S rRNA techniques.

### Bacterial Chemical Fraction Library Generation

All bacterial strains were cultured in 1 L each of YEME, R2A, and A-media in seawater (see Kyei, L. et al. 2024 for media recipes) containing HP-20 (15 g/L), XAD4 (7.5 g/L), and XAD7 (7.5 g/L) resins to capture the produced metabolites. Cultures were incubated for 7 d at 30 °C on a shaker set at 200 rpm. After 7 d, the resins from each media condition were combined, and the cultures were filtered through miracloth to recover the resins. The combined resin mixture was extracted with MeOH and acetone for up to 16 h at rt. The organic extracts were combined and dried over 4 g of Celite to produce crude extracts. These extracts were semipurified via flash chromatography using 8 g of Phenomenex Supra C8 (50 μm, 65 Å), employing a step-gradient −100% H_2_O (A −40 mL), 15% MeOH/H_2_O (B −40 mL), 30% MeOH/H_2_O (C −40 mL), 45% MeOH/H_2_O (D −40 mL), 60% MeOH/H_2_O (E −40 mL), 80% MeOH/H_2_O (F −40 mL), and 100% MeOH (G/H −80 mL). Each eluate was transferred to a 40 mL vial and mixed thoroughly. An aliquot (3 mL) of each fraction was transferred to deep 48-well plates, with each bacterial strain’s fractions arranged in one column of the plate. Plates and vials were dried under vacuum. The mass of each vials’ material was weighed upon drying to completion, and the mass of each plate’s well was then calculated using the corresponding vial mass. Each fraction in the deep 48-well plates was resuspended at 10 or 50 mg/mL, depending on feasibility. Fractions were then manually transferred to 96-well plates. Fractions initially at 50 mg/mL were subsequently diluted to 10 mg/mL. An aliquot (2 μL) of fractions A-D and E-H was added to respective LC-MS vials containing 90 μL of 50% MeCN/50% H_2_O. Next, 2 μL of this mixture was analyzed by LC-Q-TOF under the following conditions: Phenomenex Kinetex 3 μm Evo C_18_ column (2.6 × 100 mm), 0.3 mL/min, hold 30% MeCN + 0.1% formic acid (FA)/70% H_2_O + 0.1% FA for 1 min then gradient to 100% MeCN + 0.1% FA over 12.5 min and held for 1 min before re-equilibration at 30% MeCN + 0.1% FA/70% H_2_O + 0.1% FA for 4 min. MS data were collected using an Agilent 6530 Q-TOF in positive mode using Agilent MassHunter software. MS1 data were collected from 100 to 2,000 *m*/*z*. MSMS spectra were collected using data-dependent acquisition, for which the top three abundant ions in the MS1 scan were selected for fragmentation; dynamic exclusion was employed to avoid fragmenting the same ion more than twice in a 2 min range. In addition, an exclusion list was utilized that contained features that were present in the control extract of the three-nutrient media (YEME, R2A and A-media). Collision-induced dissociation was applied using a linear formula that applied a higher voltage for larger molecules [CID voltage = 10 + 0.02*­(*m*/*z*)]. MS scan rate 1/s, MSMS scan rate 3/s, source gas temperature 330 °C, gas flow 34 L/min, nebulizer 35 psig. All LC-MSMS data collected using the Agilent on the bacterial fractions are publicly available under the MassIVE accession MSV000094443.

### GNPS Molecular Networking of Bacterial Extracts

HR-MSMS data of marine isolates fraction library (264 bacterial extract fraction files, three environmental extract files, and three media blanks) was acquired as described above and previously reported.^32,33^ Files were converted to mzML format using MSConvert and uploaded to the GNPS2 platform (http://gnps2.org) for Classical Networking using the workflow classical_networking_workflow (version 2024.10.09). The data was filtered by removing all MSMS fragment ions within ±17 Da of the precursor *m*/*z*. MSMS spectra were window filtered by choosing only the top 6 fragment ions in the ±50 Da window throughout the spectrum. The precursor ion mass tolerance and MSMS fragment ion tolerance were set to 0.05 Da. A network was then created where edges were filtered to have a cosine score above 0.7 and more than 6 matched peaks. Finally, the minimum size of a molecular family was set to 2, and the maximum size was set to 100; the lowest-scoring edges were removed from molecular families until the molecular family size was below this threshold. The spectra in the network were then searched against GNPS’ spectral libraries. The library spectra were filtered in the same manner as the input data. All matches kept between network spectra and library spectra were required to have a score above 0.7 and at least 6 matched peaks.^26^ The resulting molecular network was analyzed and dereplicated through the in-browser visualizer and imported to Cytoscape for further analysis and visualization.

### Large-Scale Growth and Extraction of *B. cereus* EM195W

*B. cereus* EM195W was revived from the original isolation glycerol stocks (see above) through inoculation on an ISP2 plate. Two distinct bacterial strains were observed on the plate. Each was reisolated through subinoculation onto ISP2 plates. Once isolated, single colonies of each strain were used to start liquid cultures in YEME seawater media () and grown for 6 d (5 mL, 30 °C, 150 rpm). The cultures were then centrifuged (10 min, 4000 rpm), sterile filtered, and analyzed by LR-LCMS analysis using an Agilent 1200 series HPLC system equipped with a photodiode array detector and a Thermo LTQ mass spectrometer with an EVO C18 (Kinetex, 5 μm, 100 × 4.6 mm) under the following conditions: 0–3 min hold 10% MeCN+0.1% FA/90% H_2_O+0.1% FA, followed by a linear gradient to 100% MeCN+0.1% FA over 11 min at a flow rate of 0.3 mL/min. This confirmed that the kurstakins (retention time 10.4–12.3 min) were produced by *B. cereus* EM195W. Next, *B. cereus* EM195W was grown in 8 × 1 L of YEME in seawater containing HP-20, XAD4, and XAD7 in a 2:1:1 ratio for 9 d at 30 °C, shaking at 150 rpm to obtain and isolate enough material for NMR analysis. The resins were filtered out of the media and combined, then exhaustively extracted sequentially in MeCN, acetone, and MeOH, each for 3–16 h at rt. Chemical extracts were combined (11.1 g) and confirmed to contain the kurstakins by LR-LCMS using the method described above. The crude extract was dried under vacuum with 6 g of Celite for semipurification. The crude extract was dry loaded onto a Biotage Selekt equipped with a Phenomenex Sepra C8 (50 μm, 65 Å) column (10 g) and eluted via a step-gradient: 2 column volumes (CV) each of 100% H_2_O, 25% MeCN/75% H_2_O, 50% MeCN/50% H_2_O, 75% MeCN/25% H_2_O, and 3 CV of 100% MeCN. Each fraction was analyzed with LR-LCMS to identify fractions containing the kurstakins using the same method described above, and all such fractions (2.25 g) were combined and dried on 1 g of Celite. This semipurified extract was then further purified via flash chromatography with SiliaFlash P60 (40–63 μm, 100 mL column volume). The column was eluted using the following step-gradient: 100% hexanes, 25% EtOAc/75% hexanes, 50% EtOAc/50% hexanes, 75% EtOAc/25% hexanes, 100% EtOAc, 75% MeOH/25% EtOAc, 50% MeOH/50% EtOAc, 75% MeOH/25% EtOAc, and 100% MeOH (300 mL). Fractions were analyzed with LR-LCMS, showing that the kurstakins eluted across all 100% MeOH fractions (F9–13, 101.4 mg). F10 was used for GC-MS analysis of the tail modification studies, while F11–13 (57.3 mg) were combined for further HPLC purification of major metabolites.

### Acid Hydrolysis and Methylation of Fatty Acid Tails

Fraction 10 (3.0 mg) was treated with aqueous HCl (300 μL, 6 N, Fisher, A144C-212) at 110 °C for 16 h in a heat block. The hydrolysate was then extracted with EtOAc (0.5 mL × 3) and the organic layer was dried under vacuum. This extract was then suspended in 3:2 toluene:MeOH (300 μL) and treated with (trimethylsilyl)­diazomethane (∼100 μL, 0.6 M in hexanes, TCI, T1146) dropwise while stirring until the yellow color persisted for 30 min at rt. The reaction was then dried under vacuum to remove excess diazomethane and resuspended in EtOAc (1 mL) for GC-MS analysis. Linear chain fatty acid standards ranging from C_9–18_ (1 mg each) were combined to create one mixed-lipid standard. Branched fatty acid standards (*iso*-branched chain, odd-number carbon *anteiso*-branched chain, and even-number carbon *anteiso*-branched chain) were acquired from the Sita lab, which synthesized them as previously reported.^34^ All *iso*- and *anteiso*- standards were acquired as a mixture of varying lengths; however, fragmentation patterns were analyzed against published data for validation. Each standard was similarly treated with dropwise (trimethylsilyl)­diazomethane, dried under vacuum, and resuspended in EtOAc.

### GC-MS Analysis of Fatty Acid Tails

Methylated semicrude kurstakin tails (10 μL) and standards (2 μL each) were analyzed by GC-MS equipped with a Cyclosil B chiral column (Agilent Technologies J&W Scientific, 30 m × 0.25 mm) under the following conditions: 80 °C held for 5 min, followed by a ramp to 100 °C at 20 °C/min, a ramp to 210 °C at 1 °C/min, then a ramp to 280 °C at 35 °C/min and a hold at 280 °C for 5 min. The *iso-* and *anteiso-*branched chain fatty acid methyl esters (FAMEs) eluted at almost identical times, starting at 11.7 min for methyl 5- and 6-methyl-heptanoate ester; linear chain FAMEs eluted approximately 2–3 min after the branched chain FAMEs of the same mass, starting at 26.5 min for methyl nonanoate ester. Each peak in the F10 crude mixture was compared to the standards of the same retention time for molecular ion mass and diagnostic fragmentation patterns to confirm identity.

### HPLC Purification of the Kurstakins

Compounds **5** and **6** were purified from the normal-phase column fractions 11–13 by HPLC using a reversed-phase Synergi Hydro-RP (Phenomenex, 4 μm, 250 × 10 mm) column under the following solvent system: 0–33 min isocratic method holding 63.9% H_2_O+0.1% FA/36.1% MeCN+0.1% FA at a flow rate of 3 mL/min, tR = 10.6 min (**5**), 28.6 min (**6**). Approximately 30 mg F11–13 was processed, which yielded 4.2 mg of **5** and 8.1 mg of **6**.

#### Kurstakin 5 (**5**)

Pale yellow amorphous solid; [α]^23^_D_ −5.4 (*c* 2.8, 50% MeCN/H_2_O); ^1^H and ^13^C NMR data: see . HRMS (ESI) *m*/*z*: [M + H]^+^ Calcd for C_39_H_65_N_11_O_13_ 896.4842; Found 896.4843, Δ0.2 ppm.

#### Kurstakin 6 (**6**)

Pale yellow amorphous solid; [α]^23^_D_ −2.3 (*c* 4.9, 50% MeCN/H_2_O); ^1^H and ^13^C NMR data: see . HRMS (ESI) *m*/*z*: [M + H]^+^ Calcd for C_41_H_69_N_11_O_13_ 924.5155; Found 924.5155, Δ0.0 ppm.

### TFA Fuming of **5** and **6** NMR Samples

Compounds **5** and **6** were resuspended in *d*_6_-DMSO (150 μL) and transferred into 3 mm NMR tubes for initial ^1^H NMR spectra (64 scans) acquisition. Trifluoroacetic acid (TFA) (Thermo Scientific, 28902) fumes trapped in an LC-MS vial were bubbled through the samples within the NMR tubes with an extra-long pipet (Sigma, BR747730). The acidification was monitored by iterative ^1^H NMR experiments after each addition of TFA and continued until the exchangeable peaks had sharpened and the water peak moved past the peptide α-protons to approximately 5 ppm.

### Acid Hydrolysis and Marfey’s Analysis of Crude Extract and **5**

The configuration of amino acids present in the kurstakins was determined using Marfey’s analysis.^35^ A semicrude extract containing the kurstakins (0.75 mg) and purified **5** (0.5 mg) were each treated with aqueous HCl (300 μL, 6 N) at 110 °C for 16 h in a heat block. The hydrolysates were then dried under air and resuspended in a solution of l-FDAA (1-fluoro-2–4-dinitrophenyl-5-l-alanine amide, TCI, >98%, D2259) in acetone (1 mL; 1 mg/mL), followed by the addition of aqueous NaHCO_3_ (300 μL, 1 M, Fisher, S233-500). Authentic standards of all stereoisomers of each chiral amino acid (1 mg; l-Thr: Sigma, ≥99%, T8441; d-Thr: Thermo, 99%, B21177.06; l- *allo-*Thr: Thermo, 99%, 198541000; d-*allo-*Thr: TCI, >99%, A1518; l-Ala: Sigma, >98.5%, A7469; d-Ala: TCI, >98%, A0177; l-Ser: BeanTown Chemical, 99%, 128350; d-Ser: Sigma, ≥98%, S-4250; l-His: Sigma, ≥99.5%, 53319; d-His: TCI, >99%, H0998; l-Glu: Sigma, ≥98.5%, G8415; d-Glu: TCI, >98%, G0057) were likewise suspended in l-FDAA in acetone (1.1 mL; 1 mg/mL) and aqueous NaHCO_3_ (300 μL, 1 M). Each reaction (standards and hydrolysates) was heated to 40 °C and stirred for 1 h in a heat block. The reactions were then each quenched by the addition of aqueous HCl (300 μL, 1 M) and dried under vacuum. The reaction products were resuspended in 100 μL 50% MeCN/50% H_2_O each for LR-LCMS analysis. Derivatized standards and hydrolysate products were analyzed by LCMS using a Synergi 4 μm Hydro-RP 80Å (4.6 × 250 mm) column under the following conditions: holding 20% MeCN + 0.1% FA/80% H_2_O + 0.1% FA for 5 min, followed by a linear gradient to 40% MeCN + 0.1% FA/60% H_2_O + 0.1% FA over 85 min at a flow rate of 0.4 mL/min. The retention times of the authentic acid-l-FDAA derivatives were: l-Thr (41.1 min), d-Thr (52.2 min), l- *allo-*Thr (41.6 min), d-*allo-*Thr (45.6 min), l-Ala (57.1 min), d-Ala (67.5 min), l-Ser (37.2 min), d-Ser (38.9 min), l-His_2_ (69.9 min), d-His_2_ (77.1 min), l-Glu (48.2 min), d-Glu (52.8 min). Analysis of the retention times of our derivatized products of **5** and semicrude extract revealed peaks matching d-*allo-*Thr (45.5 min), l-Ala (57.0 min), l-Ser (37.1 min), l-His_2_ (69.9 min), l-Glu (48.2 min), and d-Glu (53.0 min), with only minor quantities of isomerization product detected for Ala, His_2_, and d-*allo-*Thr that likely arose due to the harsh reaction conditions. Extracted ion chromatograms (EIC) were generated using smoothing with Gaussian 11 and plotted in GraphPad Prism version 10.2.0.

### Sequencing and Identification of *B. cereus* EM195W

^32^ A single colony of *B. cereus* EM195W was grown in 5 mL liquid YEME to an OD_600_ of 2 (∼10^9^ cells). The dense liquid culture was centrifuged (4000 rpm; 10 min), the supernatant decanted, and the cell pellet frozen at −20 °C. DNA extraction, sequencing, and assembly took place at SeqCenter (Pittsburgh, PA, USA). DNA extraction followed the ZymoBIOMICS DNA Miniprep Kit. Illumina sequencing libraries were prepared using the tagmentation-based and PCR-based Illumina DNA Prep kit and custom IDT 10bp unique dual indices (UDI) with a target insert size of 280 bp. No additional DNA fragmentation or size selection steps were performed. Illumina sequencing was performed on an Illumina NovaSeq X Plus sequencer in one or more multiplexed shared-flow-cell runs, producing 2 × 151 bp paired-end reads. Demultiplexing, quality control and adapter trimming was performed with bcl-convert v4.2.4 (a proprietary Illumina software for the conversion of bcl files to basecalls). Short read assembly was performed with Unicycler.^36^ Assembly statistics were recorded with QUAST^37^ (versions and parameters: ). Samples were then annotated with Bakta.^38^ Species-level identification was accomplished by submission of the assembled genome to the Type (Strain) Genome Server (TYGS).^28^

### Identification of *B. cereus* EM195W and Confirmation of krs BGC

The assembled *B. cereus* EM195W genome was uploaded into AntiSMASH 8.0 ^29^ for analysis. The *krs* nonribosomal peptide synthetase (NRPS) BGC was identified by its characteristic seven-module *krs*A–C core genes. The sequence of these three core genes was aligned in BLASTn to the corresponding *krs*A–C sequence of the type genome *B. thuringiensis* 4BA1 and showed a 100% query cover, 96% identity, and <1% gaps (2/25912).

### BLASTp Identification of Strains Containing the krs BGC

Following previous methodology,^11^ the *krs*A–C genes of *B. thuringiensis* 4BA1, as identified by AntiSMASH, were translated into their amino acid sequences and copied into BLASTp. The genes were entered as one continuous sequence based on their genomic proximity in *B. thuringiensis* 4BA1; upstream and downstream genes, which were separated from *krs*A–C by noncoding gaps, were excluded. A BLASTp search against the NCBI nonredundant protein database was performed using default parameters (blastp algorithm, BLOSUM62 scoring matrix) with the following exception: the maximum target sequences was increased to 5,000 results. Results were filtered with cutoffs of ≥70% identity and ≥50% query cover, resulting in a hit list of 3115 sequences (see ). To verify the robustness of this cutoff, the genome of a dissimilar, non-*Bacillus* hit, *Thermoactinomyces* sp. DSM 45891 (GenBank assembly GCA_900119485.1, 52% identity, 72.6% identity to *B. thuringiensis* 4BA1 *krs*A–C), was analyzed in AntiSMASH to confirm the presence of the *krs* BGC. The full BLASTp sequence hit list was downloaded for analysis and visualization as a phylogenetic tree using MEGAN6 (version 6.25.10). To decrease bias in results toward *Bacillus* spp., a highly dissimilar, complete *krs*A–C sequence, *Croceifilum oryzae* DSM 46876 (NCBI accession NZ_JAUSUV010000014, 99% query cover, 73% identity to *B. thuringiensis* 4BA1 *krs*A–C), was likewise analyzed with the above workflow and visualized in MEGAN6 () for comparison to the first tree.

### Propagated FASST Searching of MSV Data Sets and Molecular Networking Results

One node of each unique kurstakin structure represented in the marine isolate molecular network (Figure 1A, 11 nodes in total) was chosen for FASST searching. Universal Spectrum Identifiers (USIs) representing each chosen node’s merged spectra were searched in the GNPS2 workflow fasst_batch_workflow (version 2014.10.09, FASST-1 step) against the database metabolomicspanrepo_index_latest with analog searching turned on. Precursor and fragment tolerances were set to 0.02, and a minimum cosine score of 0.7 was defined. MASST searching of individual USIs against all other libraries was likewise performed, broadening the results (tasks not saveable). Next, 37 USIs representing all masses and MassIVE (MSV) public data sets present in the results were chosen for a secondary “propagated” FASST search (FASST-2) using the same workflow and parameters as FASST-1. Of the 2,762 results, all duplicate USIs and spectrum files, MSVs only containing one result, and blank samples were removed, resulting in 336 unique USIs from 80 different spectrum files. These files, along with our original kurstakin-containing marine isolate files, were analyzed in GNPS2 with the classical molecular networking workflow (version 2025.01.14) using the methods described above except a minimum cosine score of 0.6 and a minimum cluster size of 1 node. Additionally, a minimum peak intensity threshold of 25 and a maximum network range of 300 Da were added. The resulting network was exported into Cytoscape and filtered to include only precursor masses of 840–1000 Da. Additionally, the cluster containing the kurstakins was filtered to exclude any nodes that did not contain the diagnostic putative decarboxylated histidine fragment (110.1 *m*/*z*).

## Supplementary Material





## Data Availability

The assembled *B. cereus* EM195W genome has been deposited at DDBJ/ENA/GenBank under the accession JBRKOR000000000. The version described in this paper is version JBRKOR010000000. The GNPS2 molecular network and FASST workflow tasks can be found here: https://gnps2.org/status?task=037f6df1ae6543468300a1d1a0bc6481 (marine isolates library molecular network), https://gnps2.org/status?task=d79005a4e22a49f78f3865ffec82b049 (FASST-1: merged spectra of Figure 1A spectral family), https://gnps2.org/status?task=25d153f0abf0466ca1f8a953a5398f48 (FASST-2: propagated FASST of FASST-1 results), https://gnps2.org/status?task=650439b14c4945bfb6365d3d7d5b8f3a (FASST results molecular network). The NMR data for the following compounds has been deposited in the Natural Products Magnetic Resonance Database (NP-MRD; www.np-mrd.org) and can be found at NP0351802 (Kurstakin 5) and NP0351778 (Kurstakin 6).

## Notes

Mass spectrometry data, NMR spectra, Marfey’s analysis, and genomic analyses (PDF)
Complete *B. thuringiensis* 4BA1 *krs*A–C BLASTp results (CSV)

## References

[1] Olishevska S., Nickzad A., Déziel E. (2019). Bacillus and
Paenibacillus Secreted
Polyketides and Peptides Involved in Controlling Human and Plant Pathogens. Appl. Microbiol. Biotechnol..

[2] Cochrane S. A., Vederas J. C. (2016). Lipopeptides from
Bacillus and Paenibacillus Spp.:
A Gold Mine of Antibiotic Candidates. Med. Res.
Rev..

[3] Upert G., Luther A., Obrecht D., Ermert P. (2021). Emerging Peptide Antibiotics
with Therapeutic Potential. Med. Drug Discovery.

[4] Straus S. K., Hancock R. E. W. (2006). Mode of Action
of the New Antibiotic for Gram-Positive
Pathogens Daptomycin: Comparison with Cationic Antimicrobial Peptides
and Lipopeptides. Biochim. Biophys. Acta.

[5] Balleux G., Höfte M., Arguelles-Arias A., Deleu M., Ongena M. (2025). Bacillus Lipopeptides
as Key Players in Rhizosphere Chemical Ecology. Trends Microbiol..

[6] Liang Z., Ali Q., Wu H., Gu Q., Liu X., Sun H., Gao X. (2025). Biocontrol Mechanism of Bacillus
Thuringiensis GBAC46 Against Diseases
and Pests Caused by Fusarium Verticillioides and Spodoptera Frugiperda. Biomolecules.

[7] Cawoy, H. ; Bettiol, W. ; Fickers, P. ; Onge, M. Bacillus-Based Biological Control of Plant Diseases. In Pesticides in the Modern World—Pesticides Use and Management; InTech, 2011. 10.5772/17184.

[8] Ongena M., Jourdan E., Adam A., Paquot M., Brans A., Joris B., Arpigny J.-L., Thonart P. (2007). Surfactin and Fengycin
Lipopeptides of Bacillus Subtilis as Elicitors of Induced Systemic
Resistance in Plants. Environ. Microbiol..

[9] Waongo B., Ndayishimiye L., Tapsoba F., Zongo W.-S. A., Li J., Savadogo A. (2025). Prospection for Potential New Non-Ribosomal Peptide
Gene Clusters in Bacillus Genus Isolated from Fermented Foods and
Soil through Genome Mining. Front. Microbiol..

[10] Pichard B., Larue J.-P., Thouvenot D. (1995). Gavaserin
and Saltavalin, New Peptide
Antibiotics Produced ByBacillus Polymyxa. FEMS
Microbiol. Lett..

[11] Béchet M., Caradec T., Hussein W., Abderrahmani A., Chollet M., Leclère V., Dubois T., Lereclus D., Pupin M., Jacques P. (2012). Structure,
Biosynthesis, and Properties
of Kurstakins, Nonribosomal Lipopeptides from Bacillus Spp. Appl. Microbiol. Biotechnol..

[12] Hathout Y., Ho Y. P., Ryzhov V., Demirev P., Fenselau C. (2000). Kurstakins:
A New Class of Lipopeptides Isolated from Bacillus Thuringiensis. J. Nat. Prod..

[13] Madonna A. J., Voorhees K. J., Taranenko N. I., Laiko V. V., Doroshenko V. M. (2003). Detection
of Cyclic Lipopeptide Biomarkers from Bacillus Species Using Atmospheric
Pressure Matrix-Assisted Laser Desorption/Ionization Mass Spectrometry. Anal. Chem..

[14] Price N. P. J., Rooney A. P., Swezey J. L., Perry E., Cohan F. M. (2007). Mass Spectrometric
Analysis of Lipopeptides from Bacillus Strains Isolated from Diverse
Geographical Locations. FEMS Microbiol. Lett..

[15] Jemil N., Hmidet N., Manresa A., Rabanal F., Nasri M. (2019). Isolation
and Characterization of Kurstakin and Surfactin Isoforms Produced
by *Enterobacter Cloacae* C3 Strain. J. Mass Spectrom..

[16] Mandal S. M., Sharma S., Pinnaka A. K., Kumari A., Korpole S. (2013). Isolation
and Characterization of Diverse Antimicrobial Lipopeptides Produced
by Citrobacter and Enterobacter. BMC Microbiol..

[17] Abderrahmani A., Tapi A., Nateche F., Chollet M., Leclère V., Wathelet B., Hacene H., Jacques P. (2011). Bioinformatics and
Molecular Approaches to Detect NRPS Genes Involved in the Biosynthesis
of Kurstakin from Bacillus Thuringiensis. Appl.
Microbiol. Biotechnol..

[18] Yu Y.-Y., Zhang Y.-Y., Wang T., Huang T.-X., Tang S.-Y., Jin Y., Mi D.-D., Zheng Y., Niu D.-D., Guo J.-H., Jiang C.-H. (2023). Kurstakin
Triggers Multicellular Behaviors in *Bacillus Cereus* AR156 and Enhances Disease Control Efficacy
against Rice Sheath Blight. Plant Dis..

[19] El
Arbi A., Rochex A., Chataigné G., Béchet M., Lecouturier D., Arnauld S., Gharsallah N., Jacques P. (2016). The Tunisian Oasis Ecosystem Is a Source of Antagonistic
Bacillus Spp. Producing Diverse Antifungal Lipopeptides. Res. Microbiol..

[20] Jamshidi-Aidji M., Dimkić I., Ristivojević P., Stanković S., Morlock G. E. (2019). Effect-Directed
Screening of Bacillus Lipopeptide Extracts
via Hyphenated High-Performance Thin-Layer Chromatography. J. Chromatogr. A.

[21] Sabaté D. C., Petroselli G., Erra-Balsells R., Carina Audisio M., Brandan C. P. (2020). Beneficial Effect of Bacillus Sp.
P12 on Soil Biological
Activities and Pathogen Control in Common Bean. Biol. Control.

[22] Trinh L. L., Le K. N., Le Lam H. A., Nguyen H. H. (2025). Cell-Free Supernatants
from Plant Growth-Promoting Rhizobacteria Bacillus Albus Strains Control
Aspergillus Flavus Disease in Peanut and Maize Seedlings. Beni-Suef Univ. J. Basic Appl. Sci..

[23] Fanaei M., Jurcic K., Emtiazi G. (2021). Detection
of Simultaneous Production
of Kurstakin, Fengycin and Surfactin Lipopeptides in Bacillus Mojavensis
Using a Novel Gel-Based Method and MALDI-TOF Spectrometry. World J. Microbiol. Biotechnol..

[24] Huarachi S. F., Petroselli G., Erra-Balsells R., Audisio M. C. (2022). Antibacterial Activity
against Enterovirulent Escherichia Coli Strains from Bacillus Amyloliquefaciens
B31 and Bacillus Subtilis Subsp. Subtilis C4: MALDI-TOF MS Profiling
and MALDI TOF/TOF MS Structural Analysis on Lipopeptides Mixtures. J. Mass Spectrom..

[25] Alemán I. M. V., Petroselli G., Erra-Balsells R., Daz M., Audisio M. C. (2024). Biotechnological
Properties of Bacillus Amylolyquefaciens B65 Isolated from an Artisanal
Tannery. World J. Microbiol. Biotechnol..

[26] Wang M., Carver J. J., Phelan V. V., Sanchez L. M., Garg N., Peng Y., Nguyen D. D., Watrous J., Kapono C. A., Luzzatto-Knaan T., Porto C., Bouslimani A., Melnik A. V., Meehan M. J., Liu W.-T., Crüsemann M., Boudreau P. D., Esquenazi E., Sandoval-Calderón M., Kersten R. D., Pace L. A., Quinn R. A., Duncan K. R., Hsu C.-C., Floros D. J., Gavilan R. G., Kleigrewe K., Northen T., Dutton R. J., Parrot D., Carlson E. E., Aigle B., Michelsen C. F., Jelsbak L., Sohlenkamp C., Pevzner P., Edlund A., McLean J., Piel J., Murphy B. T., Gerwick L., Liaw C.-C., Yang Y.-L., Humpf H.-U., Maansson M., Keyzers R. A., Sims A. C., Johnson A. R., Sidebottom A. M., Sedio B. E., Klitgaard A., Larson C. B., Boya P C. A., Torres-Mendoza D., Gonzalez D. J., Silva D. B., Marques L. M., Demarque D. P., Pociute E., O’Neill E. C., Briand E., Helfrich E. J. N., Granatosky E. A., Glukhov E., Ryffel F., Houson H., Mohimani H., Kharbush J. J., Zeng Y., Vorholt J. A., Kurita K. L., Charusanti P., McPhail K. L., Nielsen K. F., Vuong L., Elfeki M., Traxler M. F., Engene N., Koyama N., Vining O. B., Baric R., Silva R. R., Mascuch S. J., Tomasi S., Jenkins S., Macherla V., Hoffman T., Agarwal V., Williams P. G., Dai J., Neupane R., Gurr J., Rodríguez A. M. C., Lamsa A., Zhang C., Dorrestein K., Duggan B. M., Almaliti J., Allard P.-M., Phapale P., Nothias L.-F., Alexandrov T., Litaudon M., Wolfender J.-L., Kyle J. E., Metz T. O., Peryea T., Nguyen D.-T., VanLeer D., Shinn P., Jadhav A., Müller R., Waters K. M., Shi W., Liu X., Zhang L., Knight R., Jensen P. R., Palsson B. Ø., Pogliano K., Linington R. G., Gutiérrez M., Lopes N. P., Gerwick W. H., Moore B. S., Dorrestein P. C., Bandeira N. (2016). Sharing and Community Curation of Mass Spectrometry
Data with Global Natural Products Social Molecular Networking. Nat. Biotechnol..

[27] Bumpus S. B., Evans B. S., Thomas P. M., Ntai I., Kelleher N. L. (2009). A Proteomics
Approach to Discovering Natural Products and Their Biosynthetic Pathways. Nat. Biotechnol..

[28] Meier-Kolthoff J. P., Göker M. (2019). TYGS Is an
Automated High-Throughput Platform for State-of-the-Art
Genome-Based Taxonomy. Nat. Commun..

[29] Blin K., Shaw S., Vader L., Szenei J., Reitz Z. L., Augustijn H. E., Cediel-Becerra J. D.
D., de Crécy-Lagard V., Koetsier R. A., Williams S. E., Cruz-Morales P., Wongwas S., Segurado Luchsinger A.
E., Biermann F., Korenskaia A., Zdouc M. M., Meijer D., Terlouw B. R., van der Hooft J. J. J., Ziemert N., Helfrich E. J. N., Masschelein J., Corre C., Chevrette M. G., van Wezel G. P., Medema M. H., Weber T. (2025). AntiSMASH 8.0: Extended Gene Cluster
Detection Capabilities and Analyses of Chemistry, Enzymology, and
Regulation. Nucleic Acids Res..

[30] Wang M., Jarmusch A. K., Vargas F., Aksenov A. A., Gauglitz J. M., Weldon K., Petras D., da Silva R., Quinn R., Melnik A. V., van der Hooft J. J. J., Caraballo-Rodríguez A. M., Nothias L. F., Aceves C. M., Panitchpakdi M., Brown E., Di Ottavio F., Sikora N., Elijah E. O., Labarta-Bajo L., Gentry E. C., Shalapour S., Kyle K. E., Puckett S. P., Watrous J. D., Carpenter C. S., Bouslimani A., Ernst M., Swafford A. D., Zúñiga E. I., Balunas M. J., Klassen J. L., Loomba R., Knight R., Bandeira N., Dorrestein P. C. (2020). Mass Spectrometry Searches Using
MASST. Nat. Biotechnol..

[31] Mandic-Mulec I., Stefanic P., van Elsas J. D. (2015). Ecology
of Bacillaceae. Microbiol. Spectr..

[32] Kyei L., Piedl K., Menegatti C., Miller E. M., Mevers E. (2024). Discovery
of Biofilm Inhibitors from the Microbiota of Marine Egg Masses. J. Nat. Prod..

[33] Kyei L., Campbell R., Menegatti C., Mevers E. (2025). The Nobilamides: Potent
Biofilm Inhibitors Produced by the Microbiota of Moon Snail Egg Masses. ACS Omega.

[34] Kuzminski B. R. S., Burgenson W. R., Sita L. R. (2025). A Living Telomerization Process for
the Versatile and Scalable Production of ω-Substituted Fatty
Alcohols and Acids. ACS Catal..

[35] Campbell R., Kyei L., Piedl K., Zhang Z., Chen M., Mevers E. (2024). Bokeelamides: Lipopeptides from Bacteria Associated
with Marine Egg Masses. Org. Lett..

[36] Wick R. R., Judd L. M., Gorrie C. L., Holt K. E. (2017). Unicycler: Resolving
Bacterial Genome Assemblies from Short and Long Sequencing Reads. PLoS Comput. Biol..

[37] Gurevich A., Saveliev V., Vyahhi N., Tesler G. (2013). QUAST: Quality Assessment
Tool for Genome Assemblies. Bioinformatics.

[38] Schwengers O., Jelonek L., Dieckmann M. A., Beyvers S., Blom J., Goesmann A. (2021). Bakta: Rapid and Standardized Annotation of Bacterial
Genomes via Alignment-Free Sequence Identification. Microb. Genom..

