# Supplementary material for: Structural Characterization and Multiomics Analysis Reveal Extensive Diversity and Global Distribution of Kurstakin Lipopeptides
Source: J Nat Prod. 2025 Nov 27;88(12):2960–7. doi: 10.1021/acs.jnatprod.5c01212 (PMC12751101; doi:10.1021/acs.jnatprod.5c01212)
Supplement: Supplementary file 1 [file np5c01212_si_001.pdf]

SUPPORTING INFORMATION

Structural Characterization and Multi-omics Analysis Reveal Extensive Diversity and Global Distribution of Kurstakin Lipopeptides

Rose Campbell,[1] *Emily Mevers*[1,*]

[1]Department of Chemistry, Virginia Tech, Blacksburg, Virginia, 24060, USA

* Email: emevers@vt.edu

Table of Contents:

Figure S1: Key MSMS fragments of linear-peptide C_{13} kurstakin 2
Figure S2: MSMS mirror plot of linear- and cyclic-peptide C_{12} kurstakins 2
Figure S3: MSMS mirror plots of linear-peptide C_{12} and C_{13} kurstakins 3
Table S1: HR-LCMS masses for all kurstakin analogs 4
Figure S4: Stacked HR-LCMS EICs of all kurstakins in **Table S1** 4
Figure S5: GC-MS traces of all branched-chain methyl ester standards 5
Figure S6: Fragmentation of *iso* vs. *anteiso* standards 5
Figure S7: *Anteiso* kurstakins: combined EICs of diagnostic 61 neutral loss 5
Figure S8: ^{1}H NMR spectra of kurstakin 5 (**5**) with subsequent additions of TFA fumes 6
Figure S9: ^{1}H NMR spectrum of kurstakin 5 (**5**) in d_6-DMSO 6
Figure S10: gHSQC NMR spectrum of kurstakin 5 (**5**) in d_6-DMSO 7
Figure S11: H2BC NMR spectrum of kurstakin 5 (**5**) in d_6-DMSO 7
Figure S12: HMBC NMR spectrum of kurstakin 5 (**5**) in d_6-DMSO 8
Figure S13: TOCSY NMR spectrum of kurstakin 5 (**5**) in d_6-DMSO 8
Table S2: NMR assignments for kurstakin 5 (**5**) and observed correlations in d_6-DMSO 9
Figure S14: ^{1}H NMR spectrum of kurstakin 6 (**6**) in d_6-DMSO 10
Figure S15: gHSQC NMR spectrum of kurstakin 6 (**6**) in d_6-DMSO 10
Figure S16: HMBC NMR spectrum of kurstakin 6 (**6**) in d_6-DMSO 11
Figure S17: TOCSY NMR spectrum of kurstakin 6 (**6**) in d_6-DMSO 11
Table S3: NMR assignments for kurstakin 6 (**6**) and observed correlations in d_6-DMSO 12
Figure S18: Marfey's analysis results of threonine 13
Figure S19: Marfey's analysis results of alanine 13
Figure S20: Marfey's analysis results of serine 13
Figure S21: Marfey's analysis results of histidine 14
Figure S22: Marfey's analysis results of glutamate 14
Figure S23: *Krs* gene cluster in *B. cereus* EM195W identified by AntiSMASH 8.0 14
Figure S24: Phylogenetic tree of *Croceifilum oryzae* DSM 46876 *krs* BLASTp results 15
Table S4: MassIVE datasets identified to contain kurstakins by propagated FASST 15
Figure S25: Molecular network of propagated FASST results with parent masses 16
Table S5: Media Recipes 16
Table S6: Tools used by SeqCenter for genome assembly and annotation 17

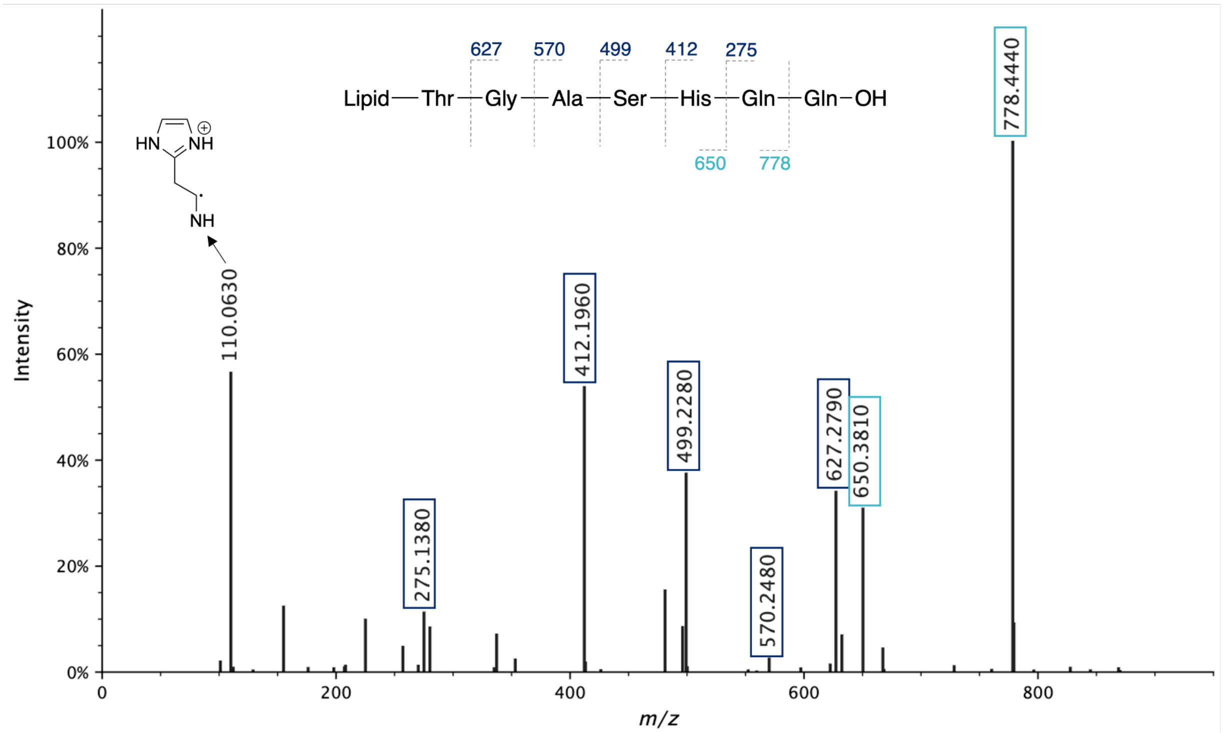

Figure S1. MSMS spectrum of linear-peptide C_{13} kurstakin showing observed *b*- (teal) and *y*-ions (navy), as well as the prominent, diagnostic decarboxylated histidine fragment. Fragments without the histidine were rarely observed, due to its basic nature.

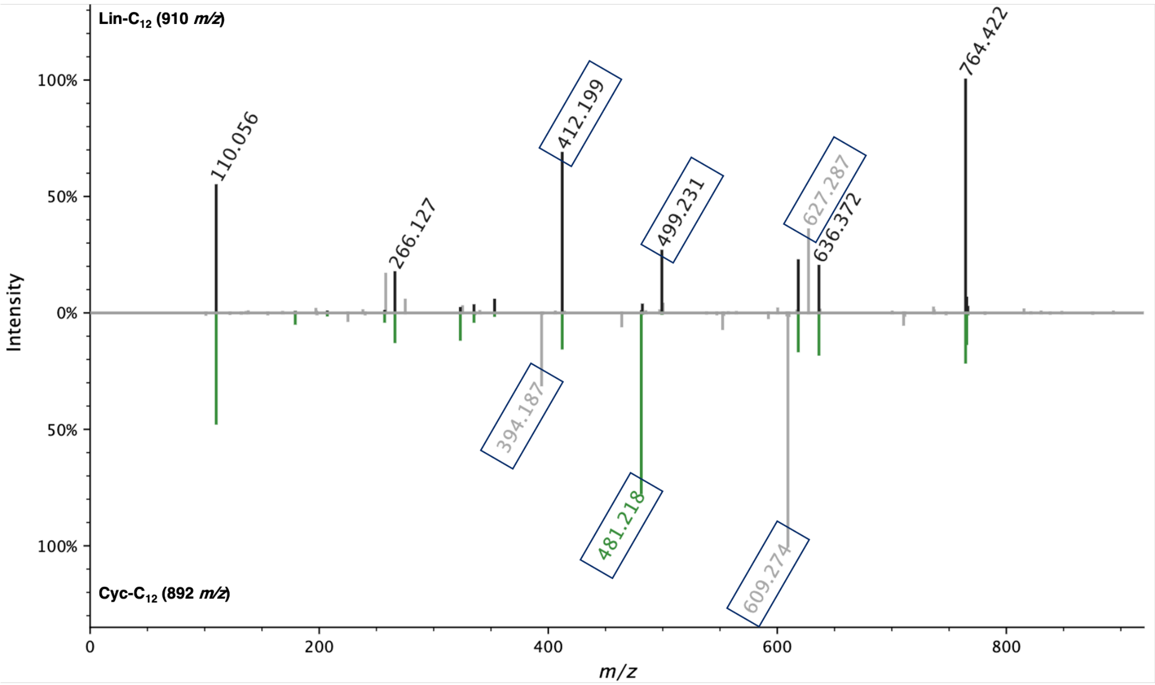

Figure S2. MSMS mirror plot of linear-peptide (top) and cyclic-peptide (bottom) C_{12} kurstakins, showing key shifted fragments containing the cyclized peptide (-18, boxed) and key common fragments not containing the terminal glutamine (unboxed).

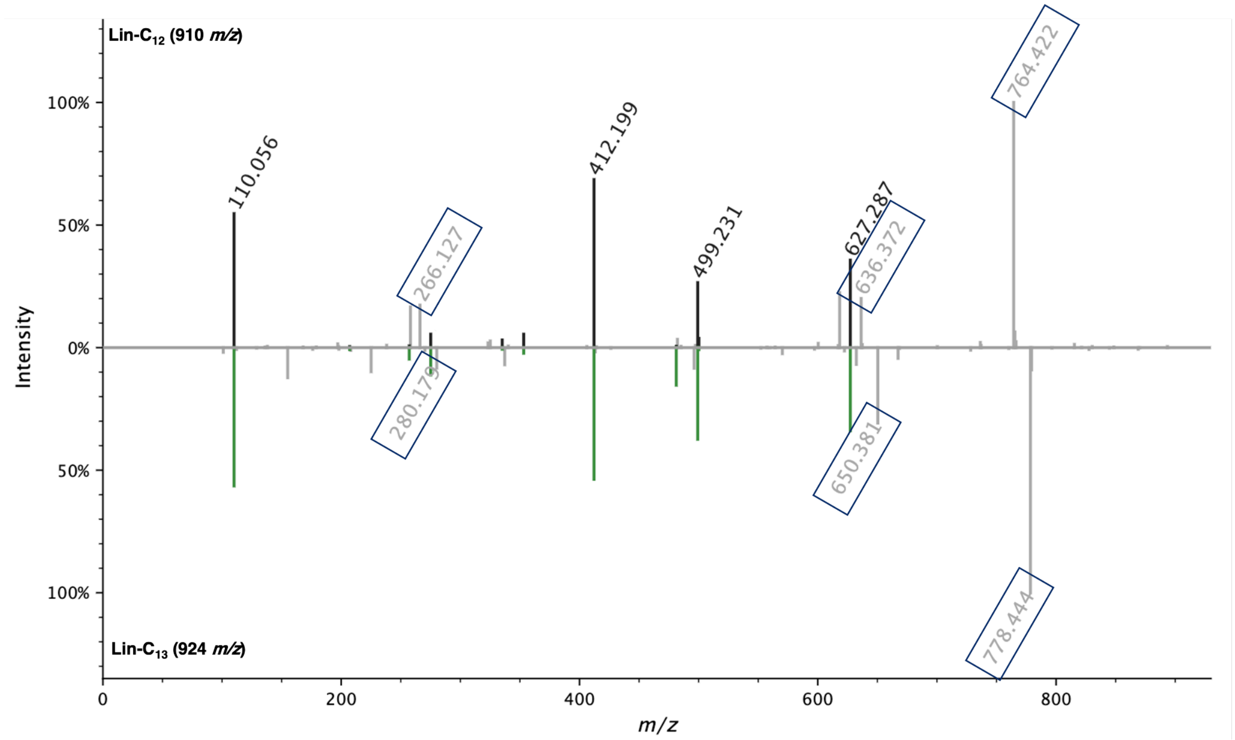

Figure S3. MSMS mirror plots of linear-peptide C_{12} (top) and C_{13} (bottom) kurstakins, showing key shifted fragments containing the lengthened tail (+14, boxed) and key common fragments containing the unmodified peptide (unboxed).

Table S1. Expected and observed HR-LCMS $[M + H]^+$ masses for all found kurstakin analogs

		Cyclic Lactone Ring Peptide				Linear Peptide			
	**Total Tail #Cs**	**Molecular Formula**	**$[M + H]^+$ Expected**	**$[M + H]^+$ Observed**	**Error (ppm)**	**Molecular Formula**	**$[M + H]^+$ Expected**	**$[M + H]^+$ Observed**	**Error (ppm)**
**Aliphatic**	C_{9}	$C_{37}H_{59}N_{11}O_{12}$	850.4423	850.4425	-0.2	$C_{37}H_{61}N_{11}O_{13}$	868.4529	868.4537	-1.0
	C_{10}	$C_{38}H_{61}N_{11}O_{12}$	864.4579	864.4581	-0.2	$C_{38}H_{63}N_{11}O_{13}$	882.4685	882.4686	-0.1
	C_{11}	$C_{39}H_{63}N_{11}O_{12}$	878.4736	878.4739	-0.4	$C_{39}H_{65}N_{11}O_{13}$	896.4842	896.4843	-0.2
	C_{12}	$C_{40}H_{65}N_{11}O_{12}$	892.4892	892.4894	-0.2	$C_{40}H_{67}N_{11}O_{13}$	910.4998	910.4999	-0.1
	C_{13}	$C_{41}H_{67}N_{11}O_{12}$	906.5049	906.5049	0.0	$C_{41}H_{69}N_{11}O_{13}$	924.5155	924.5155	0.0
	C_{14}	$C_{42}H_{69}N_{11}O_{12}$	920.5205	920.5211	-0.6	$C_{42}H_{71}N_{11}O_{13}$	938.5311	938.5313	-0.2
	C_{15}	$C_{43}H_{71}N_{11}O_{12}$	934.5362	934.5376	-1.5	$C_{43}H_{73}N_{11}O_{13}$	952.5468	952.5471	-0.4
	C_{16}					$C_{44}H_{75}N_{11}O_{13}$	966.5624	966.5623	0.1
	C_{17}					$C_{45}H_{77}N_{11}O_{13}$	980.5781	980.5766	1.5
**β-OH**	C_{9}	$C_{37}H_{59}N_{11}O_{13}$	866.4372	866.4389	-2.0				
	C_{10}	$C_{38}H_{61}N_{11}O_{13}$	880.4529	880.4521	0.9				
	C_{11}	$C_{39}H_{63}N_{11}O_{13}$	894.4685	894.4686	-0.1	$C_{39}H_{65}N_{11}O_{14}$	912.4791	912.4799	-0.9
	C_{12}	$C_{40}H_{65}N_{11}O_{13}$	908.4842	908.4843	-0.2	$C_{40}H_{67}N_{11}O_{14}$	926.4947	926.4950	-0.3
	C_{13}	$C_{41}H_{67}N_{11}O_{13}$	922.4998	922.4998	0.0	$C_{41}H_{69}N_{11}O_{14}$	940.5104	940.5104	0.0
	C_{14}	$C_{42}H_{69}N_{11}O_{13}$	936.5155	936.5161	-0.7	$C_{42}H_{71}N_{11}O_{14}$	954.5260	954.5267	-0.7
	C_{15}	$C_{43}H_{71}N_{11}O_{13}$	950.5311	950.5319	-0.8	$C_{43}H_{73}N_{11}O_{14}$	968.5417	968.5419	-0.2
	C_{16}	$C_{44}H_{73}N_{11}O_{13}$	964.5468	964.5477	-1.0	$C_{44}H_{75}N_{11}O_{14}$	982.5573	982.5573	0.0
	C_{17}					$C_{45}H_{77}N_{11}O_{14}$	996.5730	996.5715	1.5

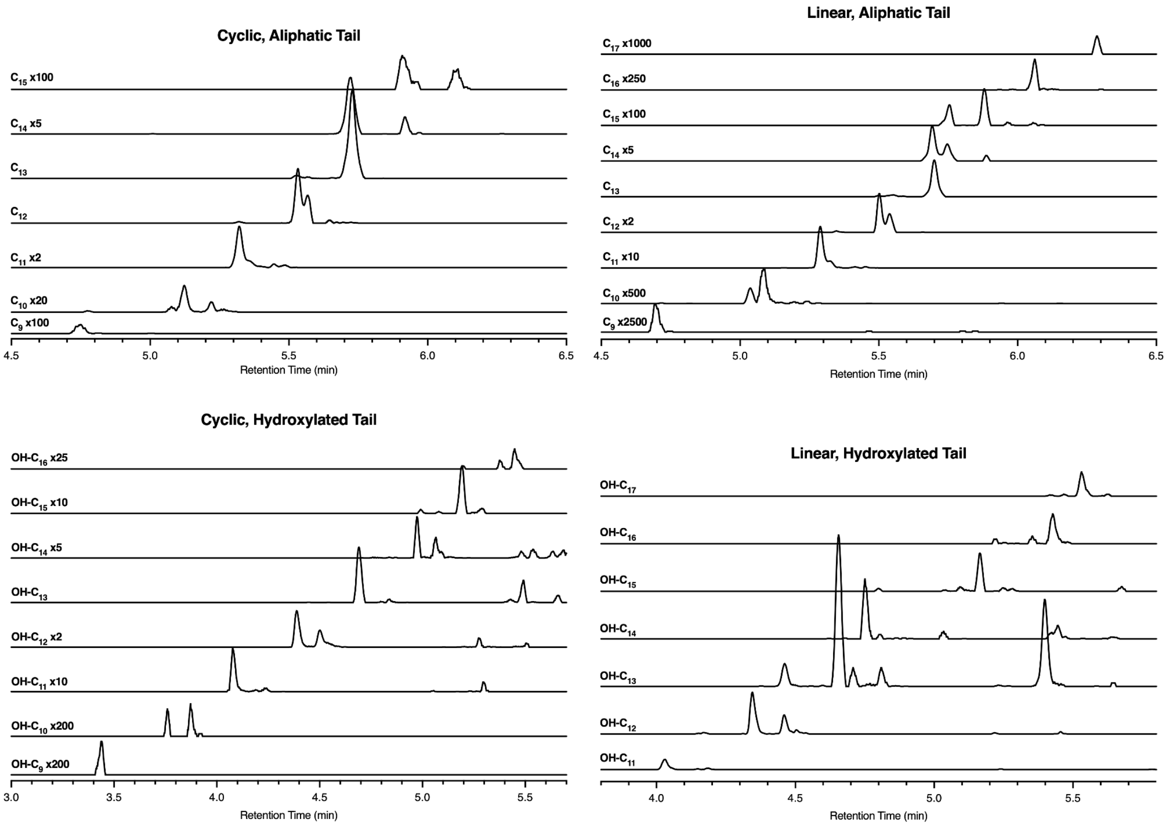

Figure S4. Stacked HR-LCMS EICs of each series of kurstakin types found in **Table S1**, scaled to show all peaks (note: extra peaks to the right side of OH-tail analog traces represent the +2 carbon isotope peak of cyclic aliphatic-tailed analogs and are not true $[M + H]^+$ peaks).

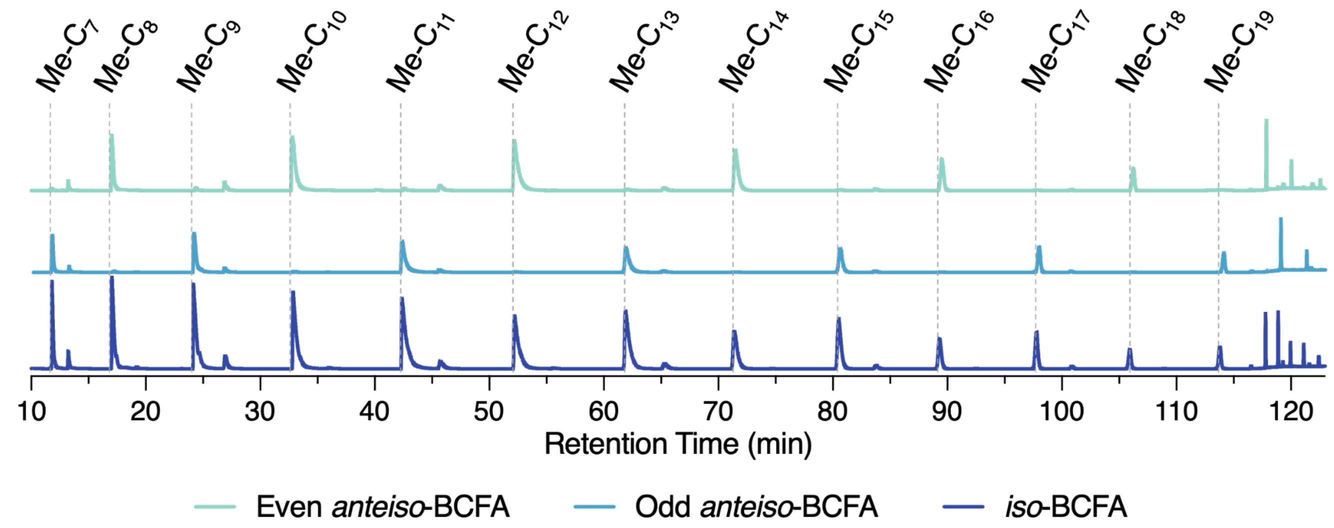

Figure S5. Stacked GC-MS traces of all branched-chain methyl ester standards (EIC of 74.1 *m/z*) showing almost identical retention times.

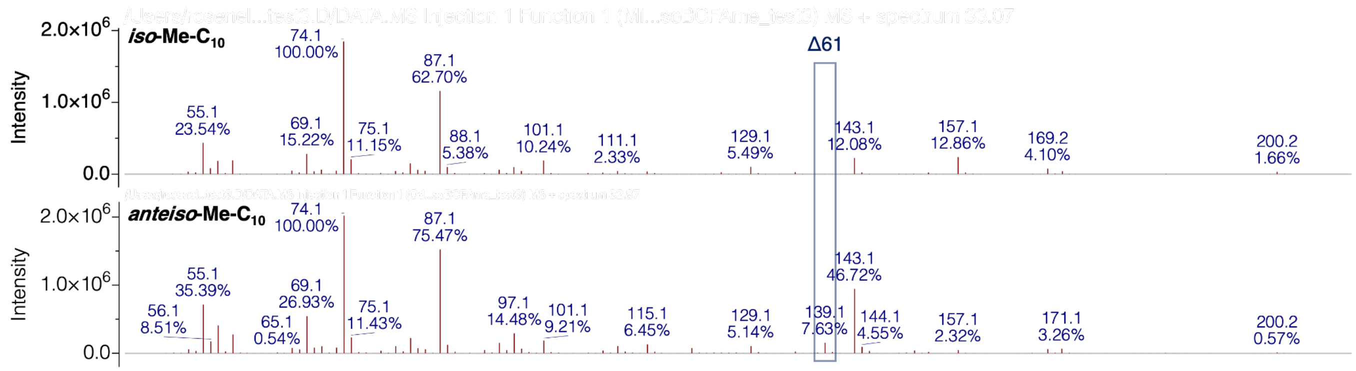

Figure S6. GC-MS fragmentation comparison of 9-Me-C_{10} methyl ester (top, *iso* configuration) and 8-Me-C_{10} methyl ester (bottom, *anteiso* configuration) standards, showing the diagnostic 61 neutral loss only present in the *anteiso* isomer.

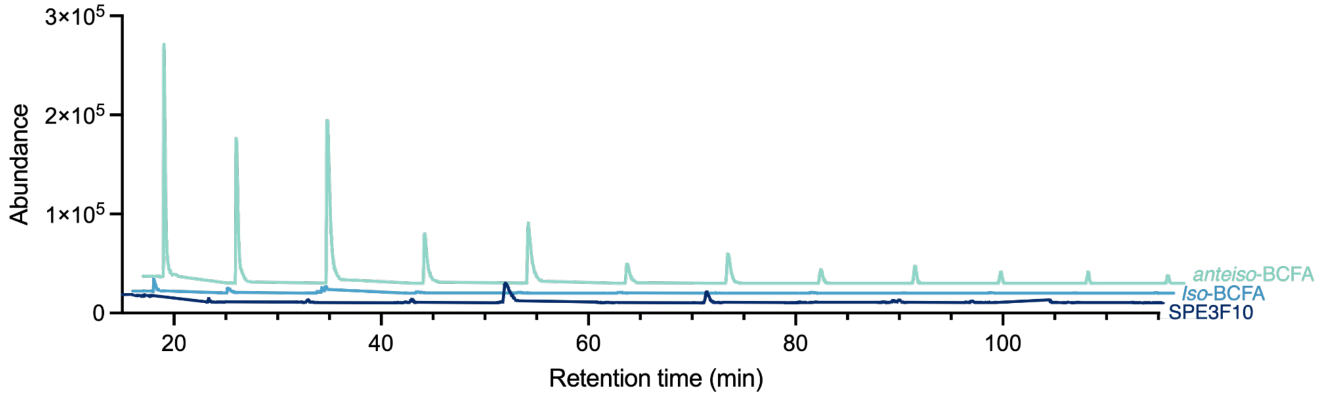

Figure S7. Combined EICs of each chain length's 61 neutral loss mass, combined as a continuous spectrum for each sample/standard, showing the presence of *anteiso*-Me-C_{12} (52.2 min) and -C_{14} (71.5 min).

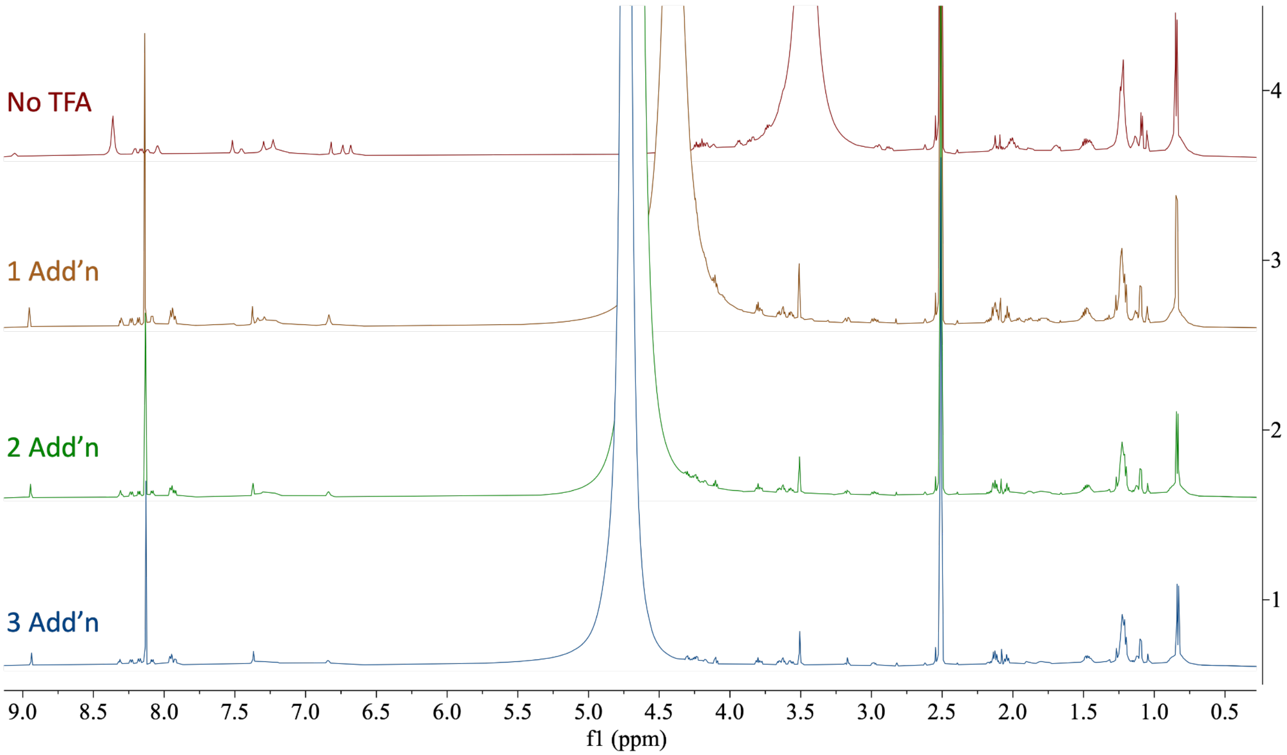

Figure S8. ^{1}H NMR spectra of kurstakin 5 (**5**) in d_6-DMSO (^{1}H 600 MHz) showing changes occurring with subsequent additions of TFA fumes

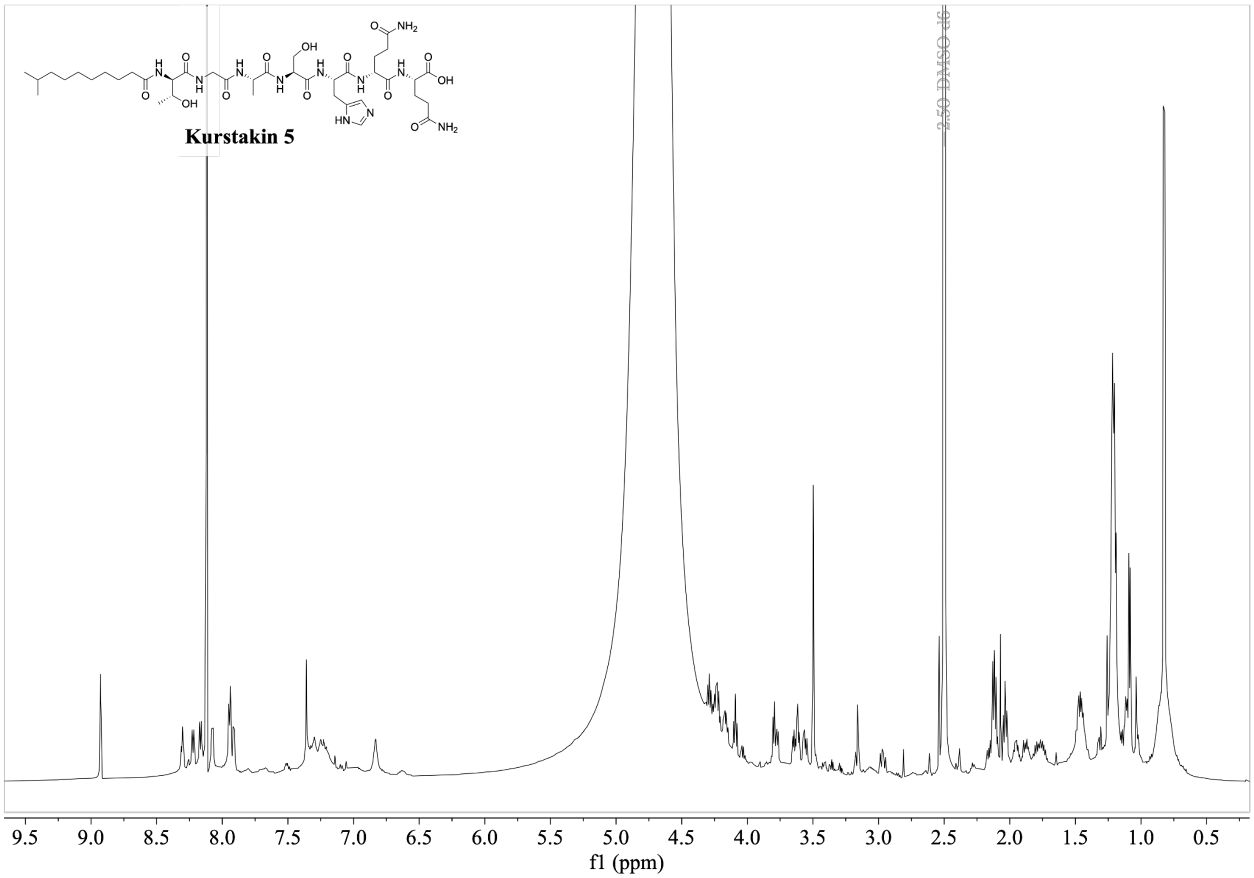

Figure S9. ^{1}H NMR spectrum of kurstakin 5 (**5**) in d_6-DMSO (^{1}H 600 MHz)

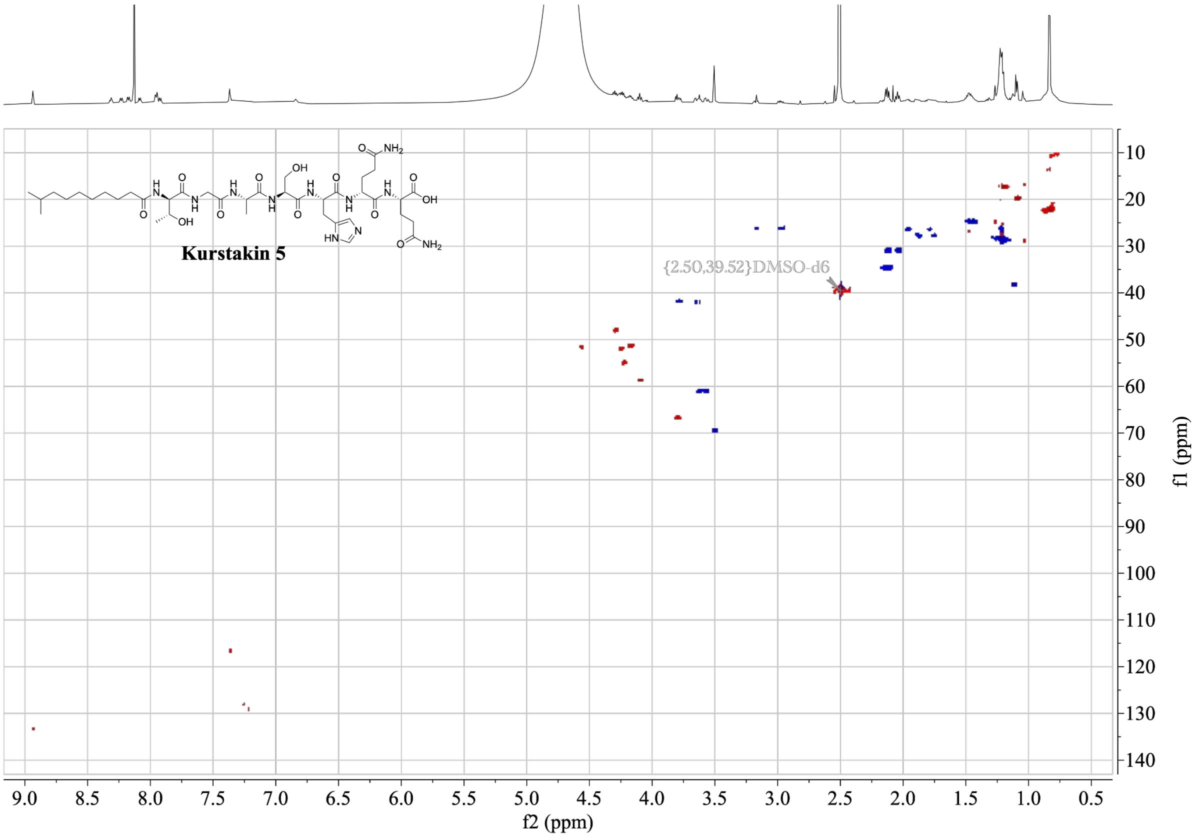

Figure S10. gHSQC NMR spectrum of kurstakin 5 (**5**) in d_6-DMSO (^{1}H 600 MHz; ^{13}C 150 MHz)

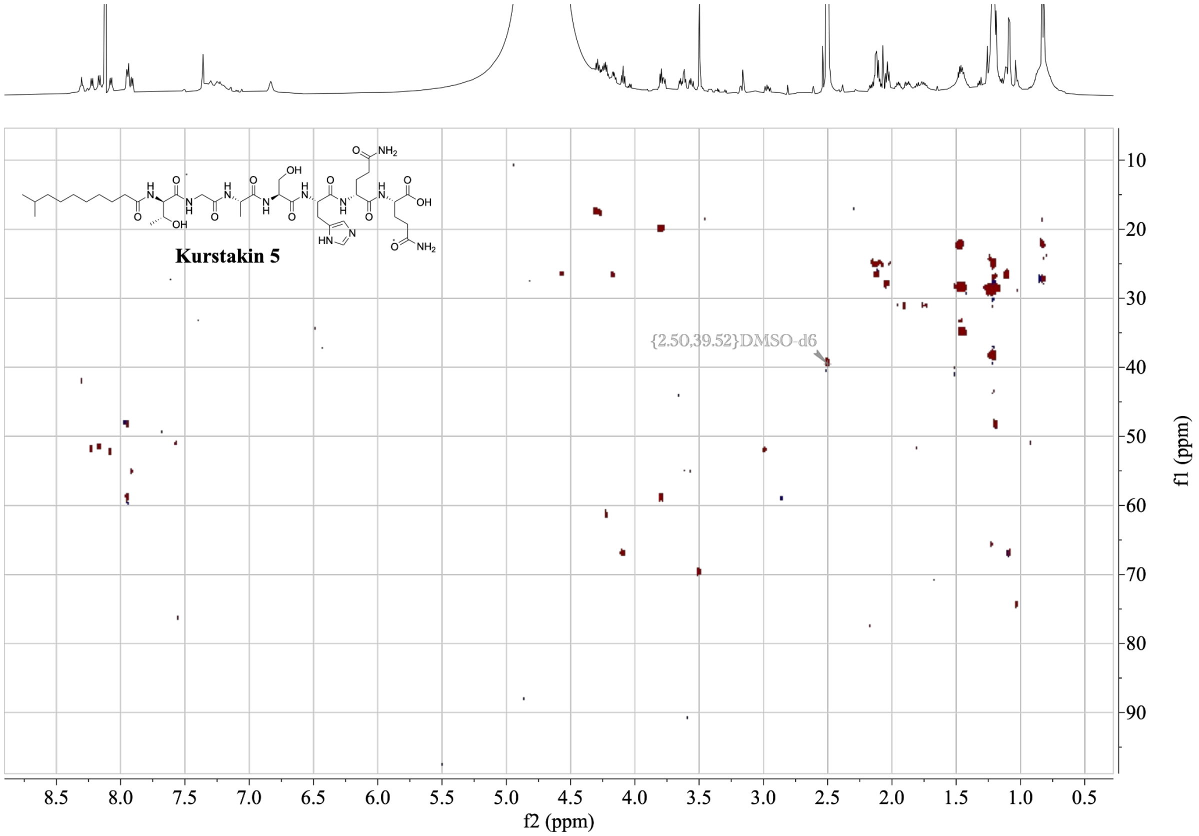

Figure S11. H2BC NMR spectrum of kurstakin 5 (**5**) in d_6-DMSO (^{1}H 600 MHz; ^{13}C 150 MHz)

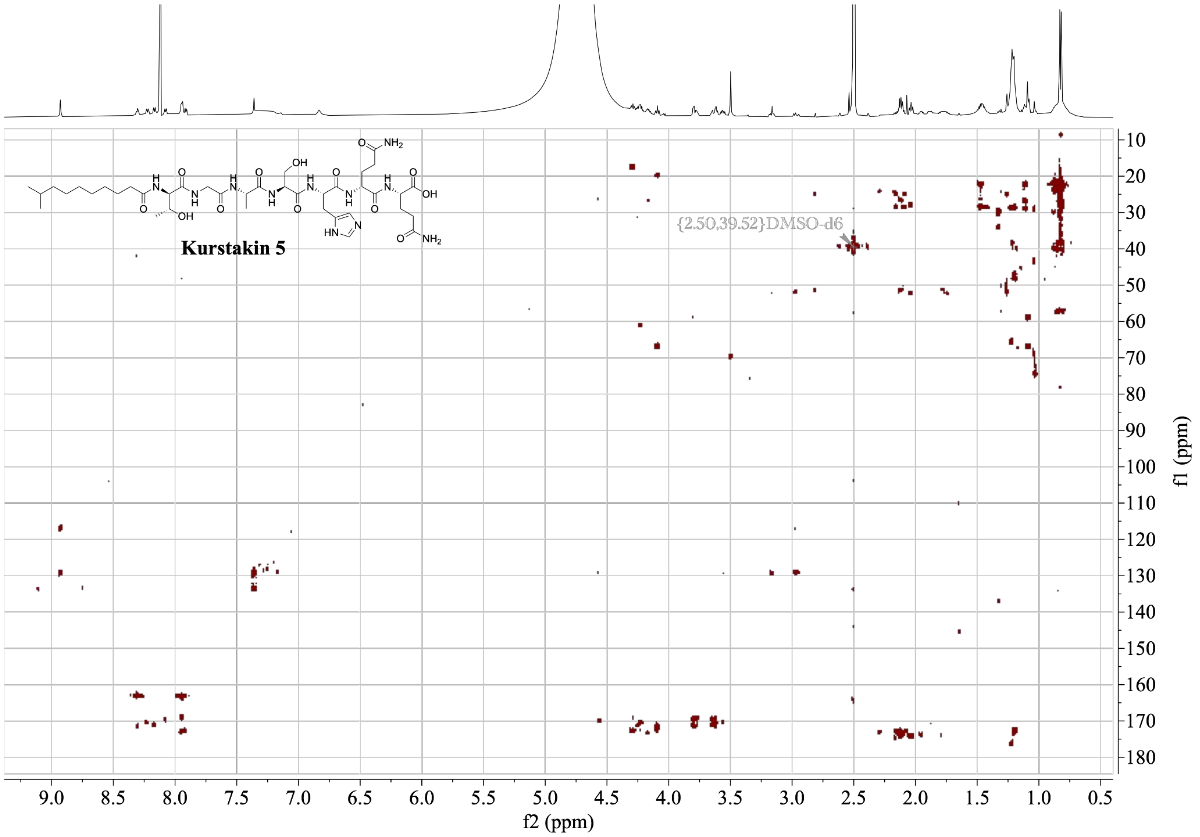

Figure S12. HMBC NMR spectrum of kurstakin 5 (**5**) in d_6-DMSO (^{1}H 600 MHz; ^{13}C 150 MHz)

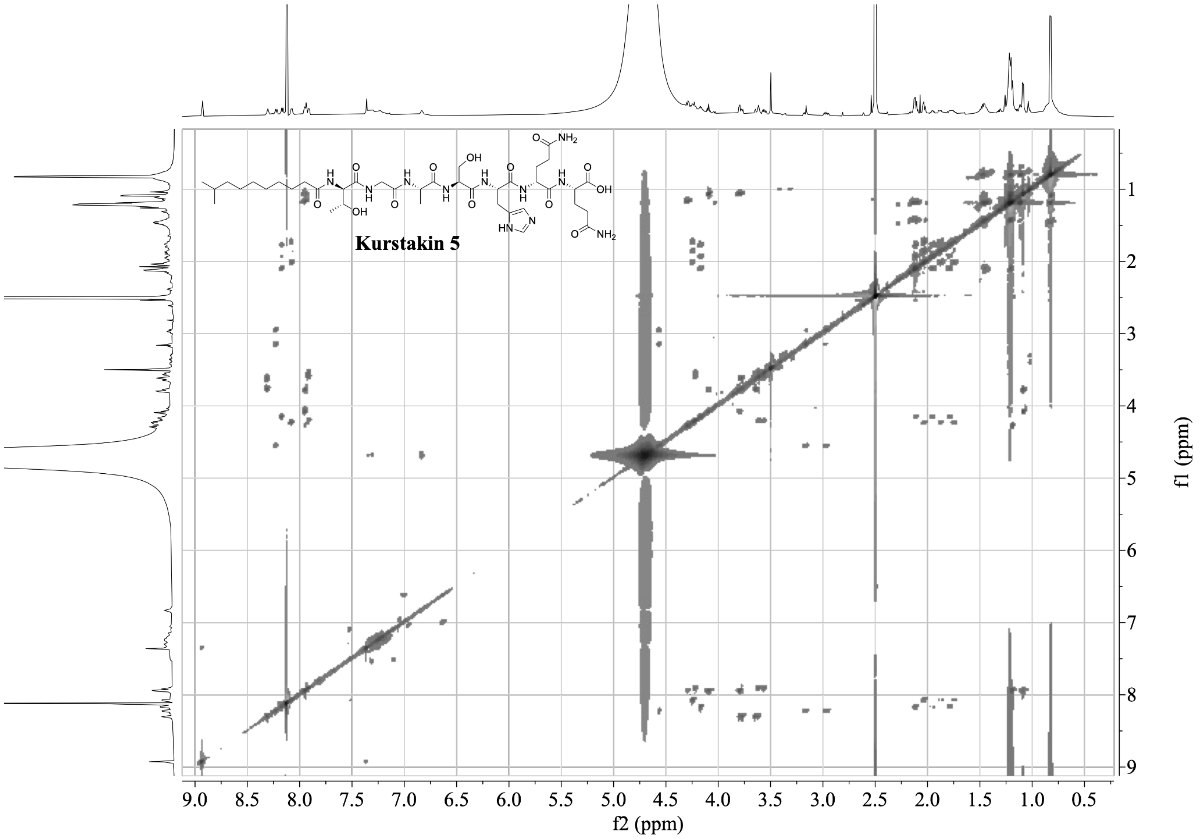

Figure S13. TOCSY NMR spectrum of kurstakin 5 (**5**) in d_6-DMSO (^{1}H 600 MHz)

Table S2. NMR assignments for kurstakin 5 (**5**) and observed correlations in d_6-DMSO

Residue	Position	∂C, type[a]	∂H (*J* in Hz)[b]	H2BC[b]	TOCSY[b]	HMBC[b]
L-Gln	1	173.3, C				
	2	51.5, CH	4.16, dt (8.4, 5.2)	3	3, 4a, 4b, 2-NH	1, 3
	3a	26.7, CH_2	1.79, m	2, 4	2, 3b, 4, 2-NH	2, 5
	3b		1.96, m	4	2, 3a, 4, 2-NH	
	4	31.1, CH_2	2.11, m	3	2, 3a, 3b, 2-NH	2, 3, 5
	5	174.0, C				
	2-NH		8.16, d (7.6)	2	2, 3a, 3b, 4	6
D-Gln	6	171.1, C				
	7	52.3, CH	4.24, m	8	8, 9a, 9b, 7-NH	6, 9
	8a	28.0, CH_2	1.75, m	7, 9	7, 8b, 9, 7-NH	7
	8b		1.88, m	9	7, 8a, 9 7-NH	
	9	31.2, CH_2	2.04, t (8.0)	8	7, 8a, 8b, 7-NH	7, 8, 10
	10	174.1, C				
	7-NH		8.08, d (7.7)	7	7, 8a, 8b, 9	11
L-His	11	169.7, C				
	12	51.8, CH	4.56, m	13	13a, 13b, 12-NH	11, 13, 14
	13a	26.4, CH_2	2.96, dd (15.1, 8.8)	12	12, 13b, 12-NH	12, 14
	13b		3.18, dd (14.7, 5.3)		12, 13a, 12-NH	12, 14
	14	129.4, C				
	15	116.9, CH	7.36, s		16	14, 16
	16	133.5, CH	8.93, s		15	14, 15
	12-NH		8.22, d (7.7)	12	12, 13a, 13b	17
L-Ser	17	170.4, C				
	18	55.1, CH	4.22, m	19	19a, 19b, 18-NH	17, 19, 20
	19a	61.2, CH_2	3.57, dd (10.9, 6.0)	18	18, 19b, 18-NH	17
	19b		3.62, m	18	18, 19a, 18-NH	17
	18-NH		7.91, d (7.1)	18	18, 19a, 19b	20
L-Ala	20	172.7, C				
	21	48.2, CH	4.29, quint (7.1)	22	22, 21-NH	20, 22, 23
	22	17.5, CH_3	1.19, m	21	21, 21-NH	20, 21
	21-NH		7.939, m	21	21, 22	21, 23
Gly	23	169.2, C				
	24a	42.2, CH_2	3.64, m		24b, 24-NH	23, 25
	24b		3.78, m		24a, 24-NH	23, 25
	24-NH		8.30, t (5.6)	24	24a, 24b	24, 25
D-*allo*-Thr	25	171.7, C				
	26	58.9, CH	4.09, t (7.7)	27	27, 28, 26-NH	25, 27, 28, 29
	27	66.8, CH	3.79, m	26, 28	26, 28, 26-NH	26
	28	20.0, CH_3	1.09, d (6.2)	27	26, 27, 26-NH	26, 27
	26-NH		7.943, m	26	26, 27, 28	29, 27
FA Tail	29	173.0, C				
	30a	34.9, CH_2	2.10, m	31	30b, 31, 32-34	29, 31, 32-34
	30b		2.15, m	31	30a, 31, 32-34	29, 31, 32-34
	31	25.0, CH_2	1.45, m	30, 32-34	30a, 30b, 32-35	32-34
	32-34[c]	28.7, CH_2	1.22, m	31, ea. other, 35	30a, 31, 35, 36	Ea. other, 35
	35	26.7, CH_2	1.21, m	36, 32-34	32-34, 36, 37, 38/39	32-34, 36
	36	38.4, CH_2	1.11, m	35	32-35, 37, 38/39	32-34, 37, 38/39
	37	27.2, CH	1.47, m	36, 38/39	32-35, 36, 38/39	32-34, 35, 36, 38/39
	38/39	22.4, CH_3	0.82, d (6.6)	37	35, 36, 37	Ea. other, 36, 37

[a]150 MHz for ^{13}C NMR; [b]600 MHz for ^{1}H NMR, H2BC, HMBC, and TOCSY; [c]CH_2 envelope

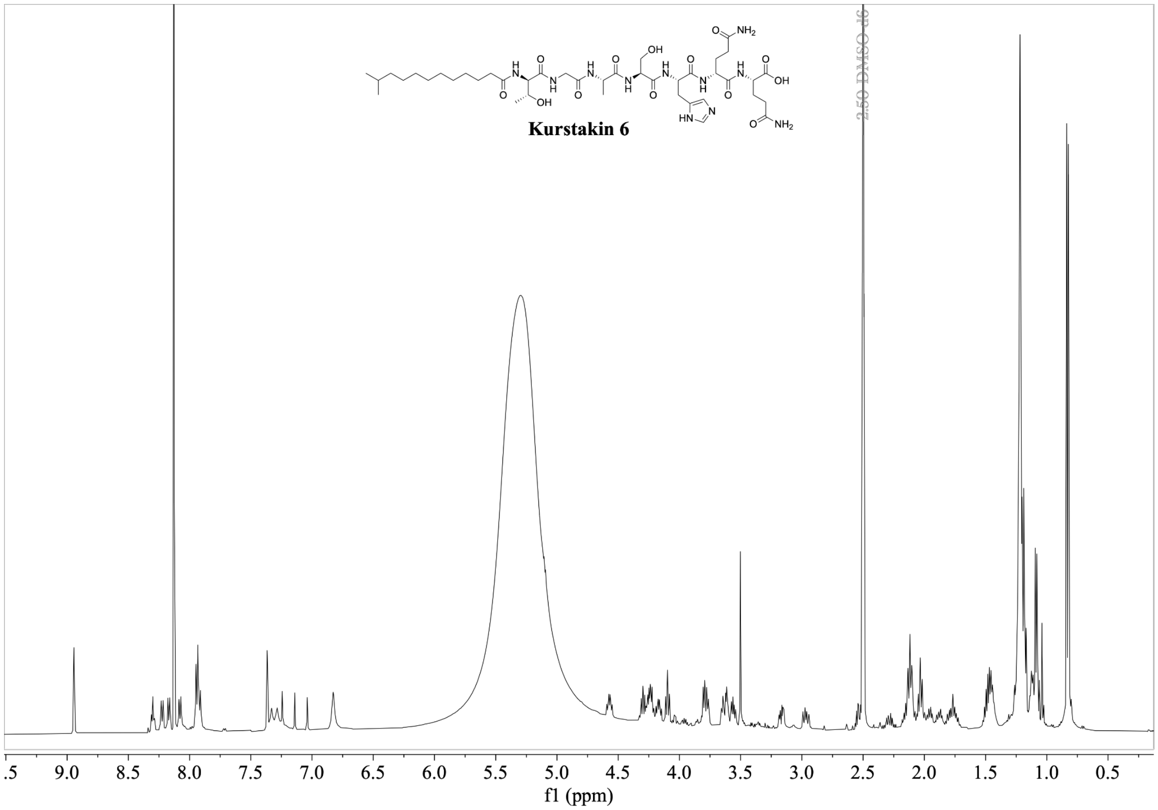

Figure S14. ^{1}H NMR spectrum of kurstakin 6 (**6**) in d_6-DMSO (^{1}H 500 MHz)

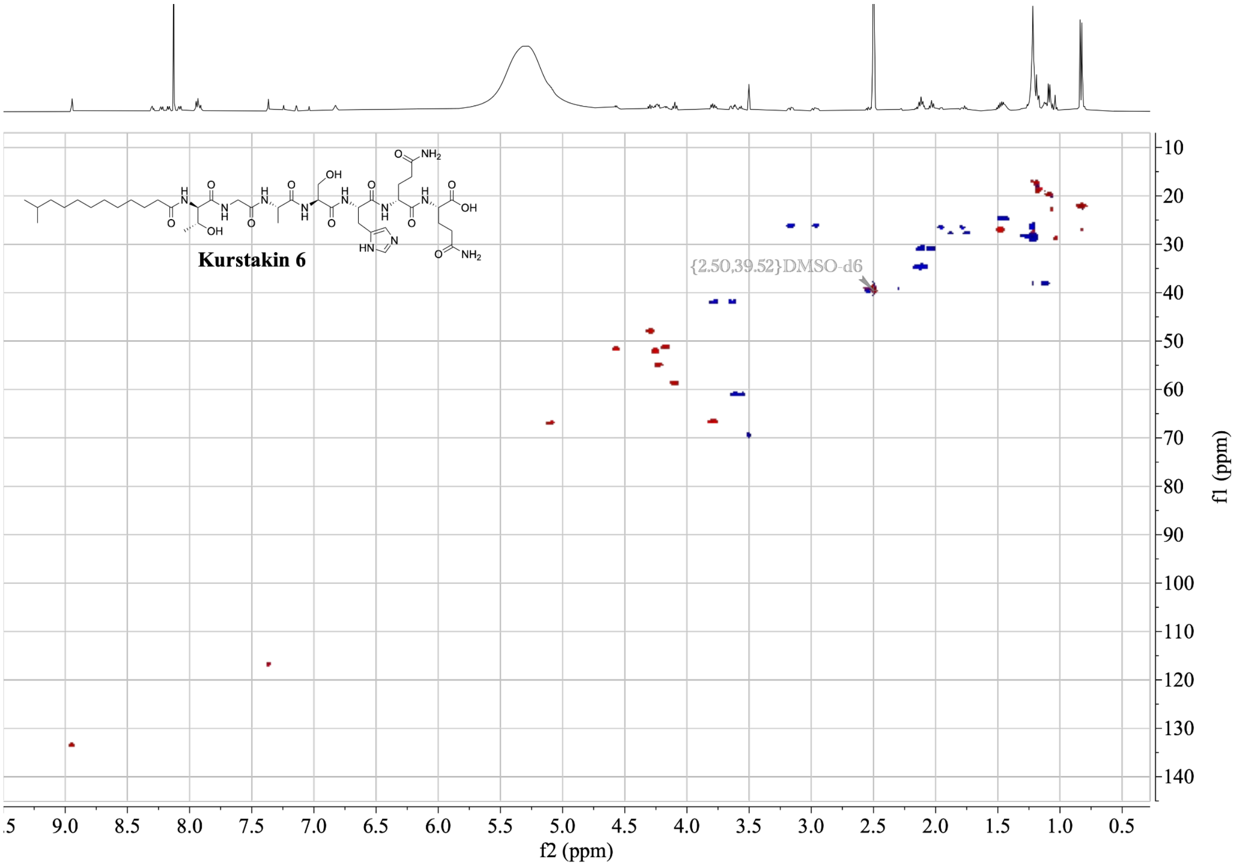

Figure S15. gHSQC NMR spectrum of kurstakin 6 (**6**) in d_6-DMSO (^{1}H 500 MHz; ^{13}C 125 MHz)

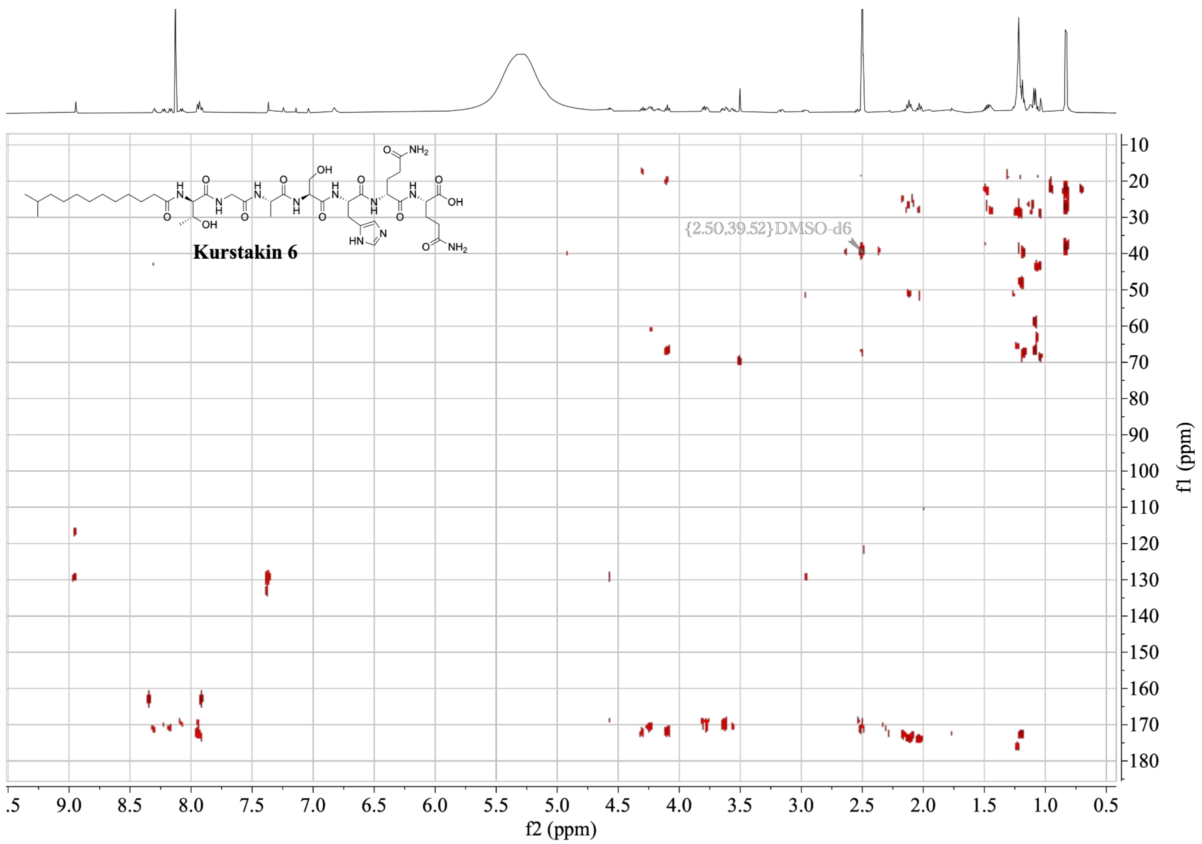

Figure S16. HMBC NMR spectrum of kurstakin 6 (**6**) in d_6-DMSO (^{1}H 500 MHz; ^{13}C 125 MHz)

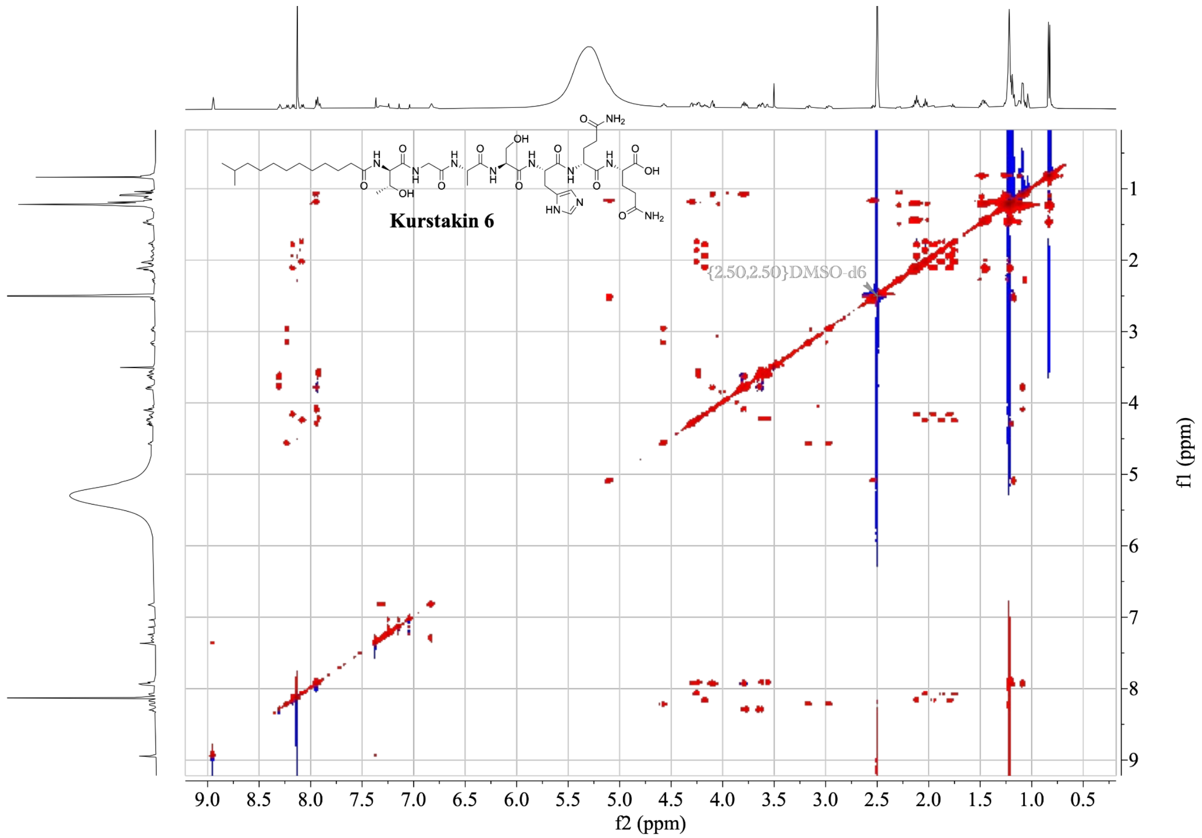

Figure S17. TOCSY NMR spectrum of kurstakin 6 (**6**) in d_6-DMSO (^{1}H 500 MHz)

Table S3. NMR assignments for kurstakin 6 (**6**) and observed correlations in d_6-DMSO

Residue	Position	∂_C, type[a]	∂_H (J in Hz)[b]	TOCSY[b]	HMBC[b]
L-Gln	1	172.8, C			
	2	51.4, CH	4.17, m	3a, 3b, 4, 2-NH	1
	3a	26.7, CH_2	1.79, m	2, 3b, 4, 2-NH	5
	3b		1.96, m	2, 3a, 4, 2-NH	
	4	31.0, CH_2	2.12, m	2, 3a, 3b, 2-NH	1, 2
	5	175.1, C			
	2-NH		8.17, d (7.7)	2, 3a, 3b, 4	6
D-Gln	6	170.7, C			
	7	52.2, CH	4.25, m	8, 9a, 9b, 7-NH	6
	8a	27.9, CH_2	1.76, m	7, 8b, 9, 7-NH	10
	8b		1.88, m	7, 8a, 9, 7-NH	
	9	31.1, CH_2	2.03, m	7, 8a, 8b, 7-NH	7
	10	172.2, C			
	7-NH		8.08, d (7.7)	7, 8a, 8b, 9	11
L-His	11	169.0, C			
	12	51.8, CH	4.57, q (7.2)	13a, 13b, 12-NH	11, 14
	13a	26.5, CH_2	2.96, dd (15.3, 8.6)	12, 13b, 12-NH	12, 14
	13b		3.16, dd (15.2, 5.0)	12, 13a, 12-NH	
	14	129.4, C			
	15	116.8, CH	7.37, s	16	14, 16
	16	133.5, CH	8.94, s	15	14, 15
	12-NH		8.22, d (7.7)	12, 13a, 13b	17
L-Ser	17	170.3, C			
	18	55.1, CH	4.23, m	19a, 19b, 18-NH	17, 19
	19a	61.2, CH_2	3.56, dd (10.9, 6.0)	18, 19b, 18-NH	17
	19b		3.61, m	18, 19a, 18-NH	17
	18-NH		7.92, m	18, 19a, 19b	20
L-Ala	20	172.7, C			
	21	48.1, CH	4.30, quint (7.1)	22, 21-NH	20, 22
	22	17.7, CH_3	1.20, m	21, 21-NH	20, 21
	21-NH		7.939, m	21, 22	23
Gly	23	170.2, C			
	24a	42.1, CH_2	3.65, m	24b, 24-NH	23
	24b		3.77, m	24a, 24-NH	23
	24-NH		8.30, t (5.8)	24a, 24b	24, 25
D-*allo*-Thr	25	171.6, C			
	26	58.8, CH	4.10, t (7.7)	27, 28, 26-NH	25, 27, 28
	27	66.8, CH	3.79, m	26, 28, 26-NH	25
	28	19.9, CH_3	1.09, d (6.2)	26, 27, 26-NH	26, 27
	26-NH		7.942, m	26, 27, 28	29
FA Tail	29	173.5, C			
	30a	34.9, CH_2	2.01, m	31, 32-36	29, 31
	30b		2.13, m	31, 32-36	29, 31
	31	25.0, CH_2	1.45, m	30a, 30b, 32-36	32-36
	32-36[c]	28.7, CH_2	1.22, m	30b, 31, 37, 38	Ea. other, 37, 38
	37	26.6, CH_2	1.21, m	32-36, 38, 39, 40/41	32-36
	38	38.3, CH_2	1.12, m	32-36, 37, 39, 40/41	37, 39
	39	27.2, CH	1.48, m	32-36, 37, 38, 40/41	40/41
	40/41	22.4, CH_3	0.83, d (6.6)	37, 38, 39	Ea. other, 38, 39

[a]125 MHz for ^{13}C NMR; [b]500 MHz for ^{1}H NMR, HMBC, and TOCSY; [c]CH_2 envelope

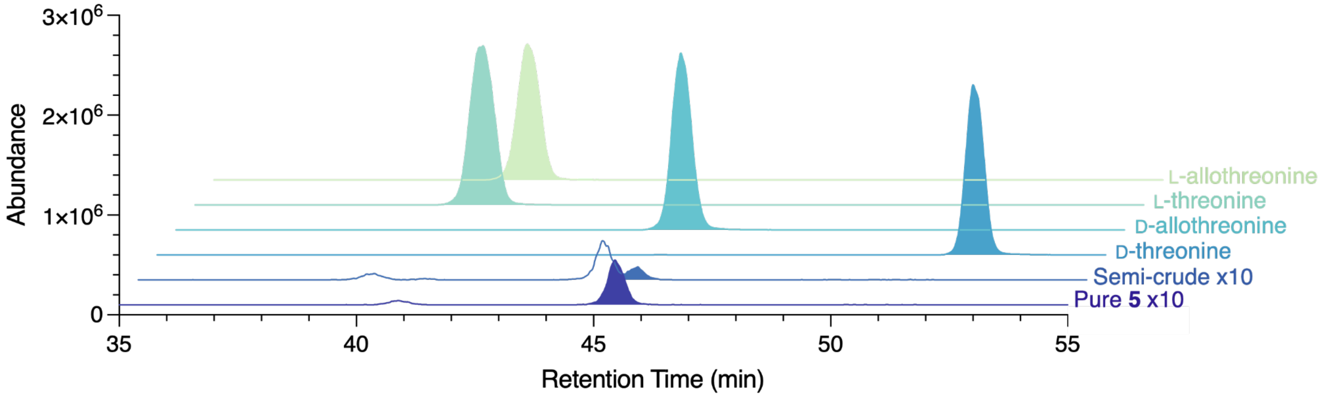

Figure S18. Marfey's analysis results of Thr (EIC of 372.1 *m/z*) showing the presence of D-*allo*-Thr. The abundance of the semi-crude and pure chromatograms is multiplied by 10 for easier visualization.

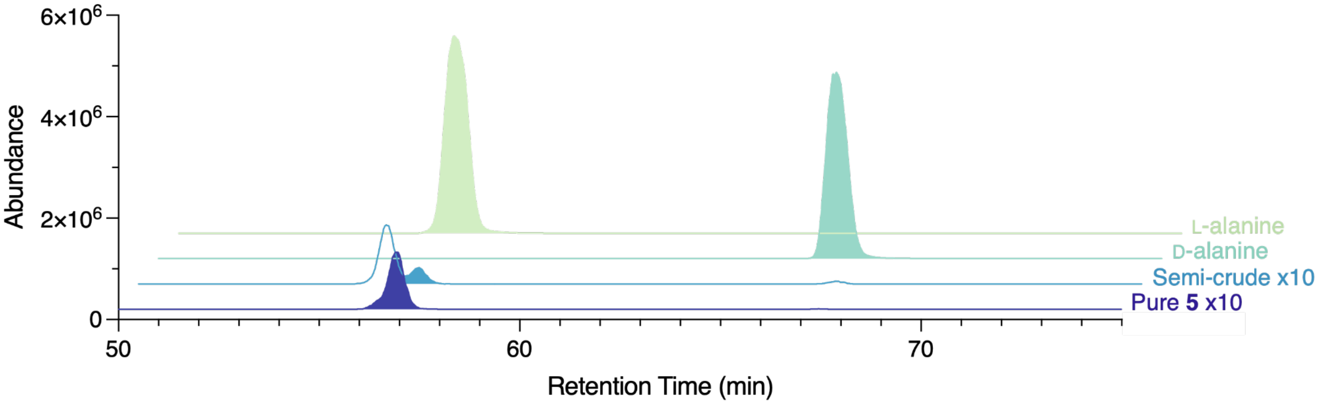

Figure S19. Marfey's analysis results of Ala (EIC of 342.1 *m/z*) showing the presence of L-Ala. The abundance of the semi-crude and pure chromatograms is multiplied by 10 for easier visualization.

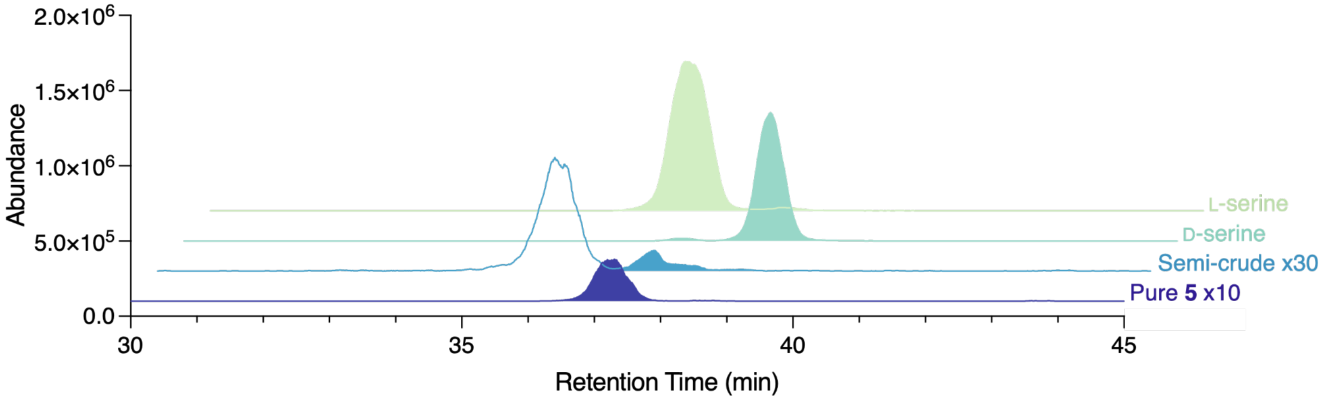

Figure S20. Marfey's analysis results of Ser (EIC of 358.1 *m/z*) showing the presence of L-Ser. The abundance of the semi-crude and pure chromatograms is multiplied by 30and 10, respectively, for easier visualization.

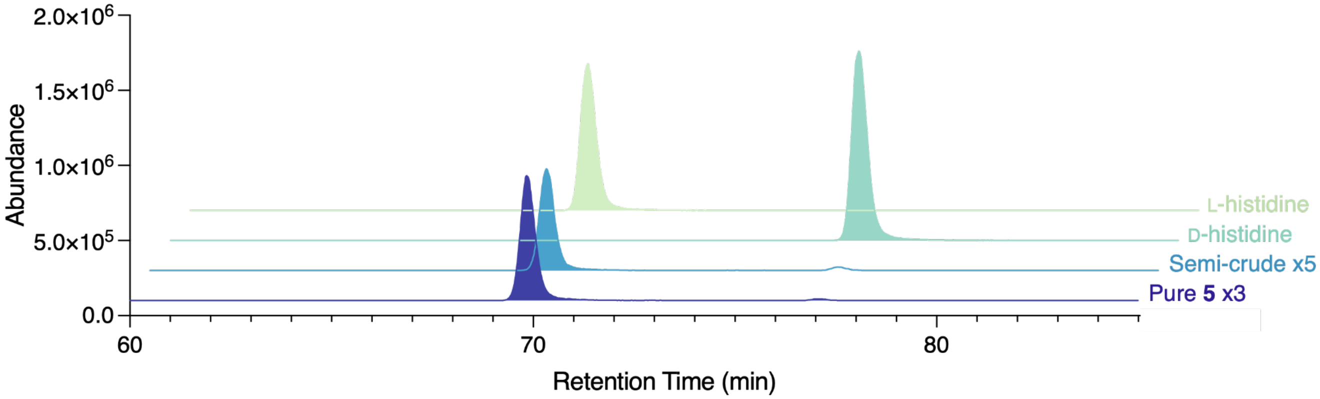

Figure S21. Marfey's analysis results of His [(FDAA)$_2$-His, EIC of 660.2 *m/z*] showing the presence of L-His. The abundance of the semi-crude and pure chromatograms is multiplied by 5 and 3, respectively, for easier visualization.

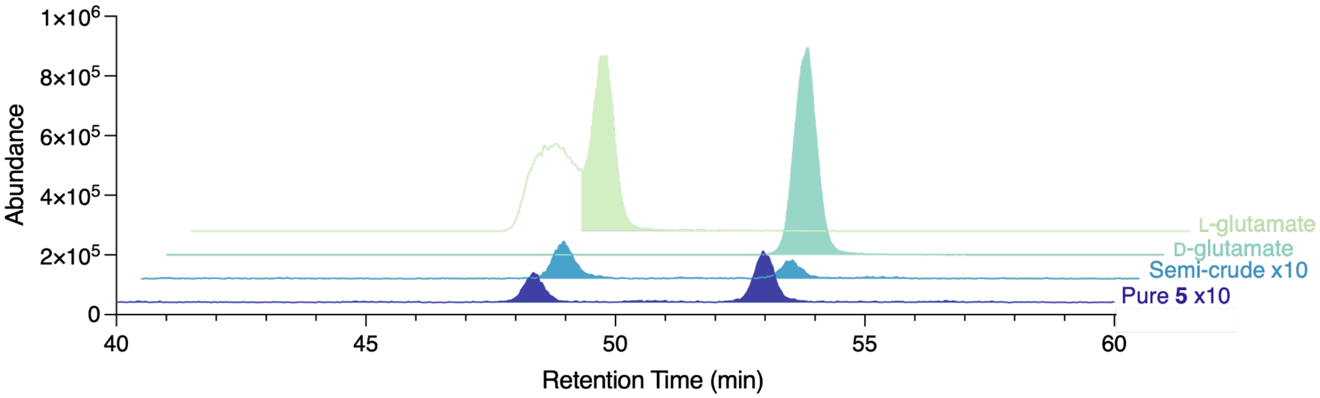

Figure S22. Marfey's analysis results of Glu (EIC of 400.1 *m/z*) showing the presence of both L- and D-Gln. The abundance of the semi-crude and pure chromatograms is multiplied by 10 for easier visualization.

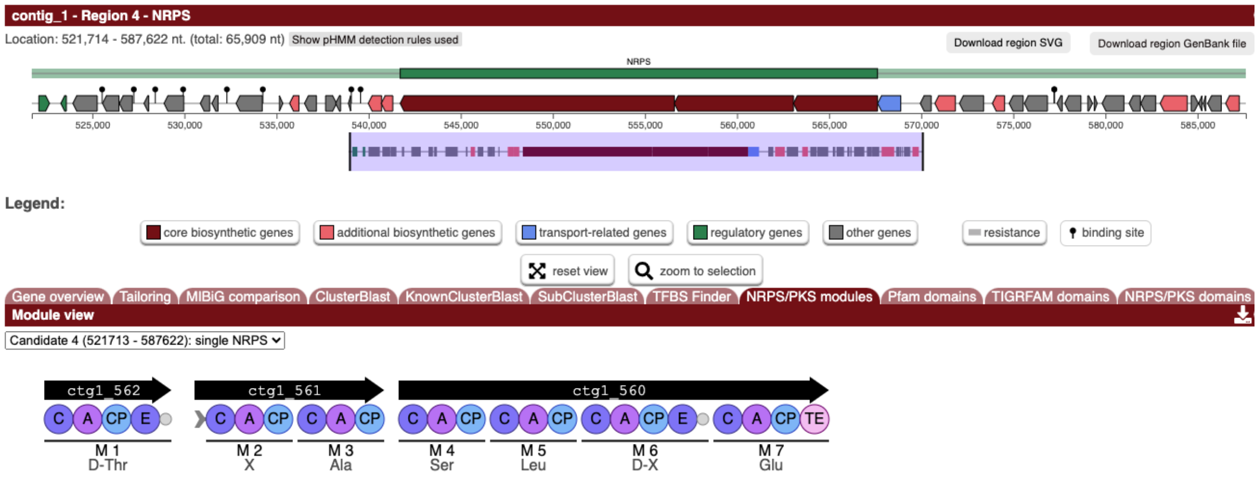

Figure S23. *Krs* gene cluster in *B. cereus* EM195W identified by AntiSMASH 8.0, with epimerization domains in the expected modules 1 and 6, and predicted adenylation domain specificity closely matching predictions of known *krs* BGCs.

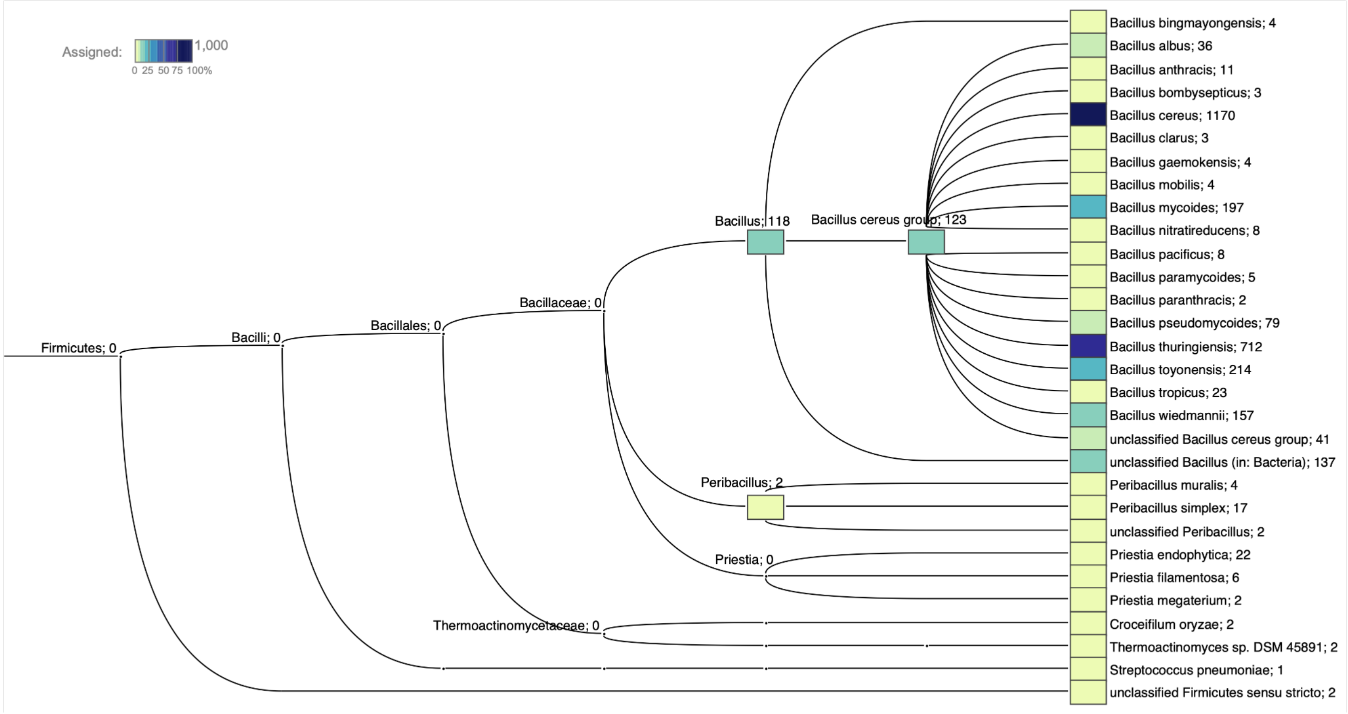

Figure S24. Phylogenetic tree of bacterial species containing the *krs* BGC that aligned with ≥70% identity and ≥50% query cover in BLASTp to *krs*A-C in *Croceifilum oryzae* DSM 46876.

Table S4. MassIVE datasets identified to contain kurstakins by propagated FASST searching

MSV Dataset	Description
94443	QUERY DATA: Bacterial isolates from marine egg masses
80922	Bacterial isolates from multiple cave sites
81381	Bacterial isolates from *Arabidopsis thaliana* roots and shoots extracted and used in binary assays
81671	*Trachymyrmex septentrionalis* fungus garden samples, collected from New Jersey, Georgia, North Carolina and Florida
82081	*Bacillus subtilis* strains that either had or hadn't interacted with fungi
84024	Extracted *Trachymyrmex septentrionalis* fungus garden samples
84951	28 soil bacteria isolated from Wisconsin
86550	Bacteria isolated from human skin
87793	*Burkholderia cenocepacia* (3 clinical isolates) grown with and without antibiotic stress
88196	Bacterial isolates from populus trees
89255	*Burkholderia cenocepacia* (2 strains) knockout studies
90888	*Bacillus subtilis* NCIB3610, *B. cereus* EA-CB1047 and *B. tequilensis* EA-CB0015 grown with & without triphenyl tetrazolium chloride
93329	83 strains of Actinomycetes
94644	*Methylobacterium* sp. Leaf119 grown with & without isotope labeling and PABA/methionine feeding
95148	Bacterial isolates from a plant microbiome
95439	Methanotrophs cocultured
95664	*Bacillus* spp. induced metabolite studies

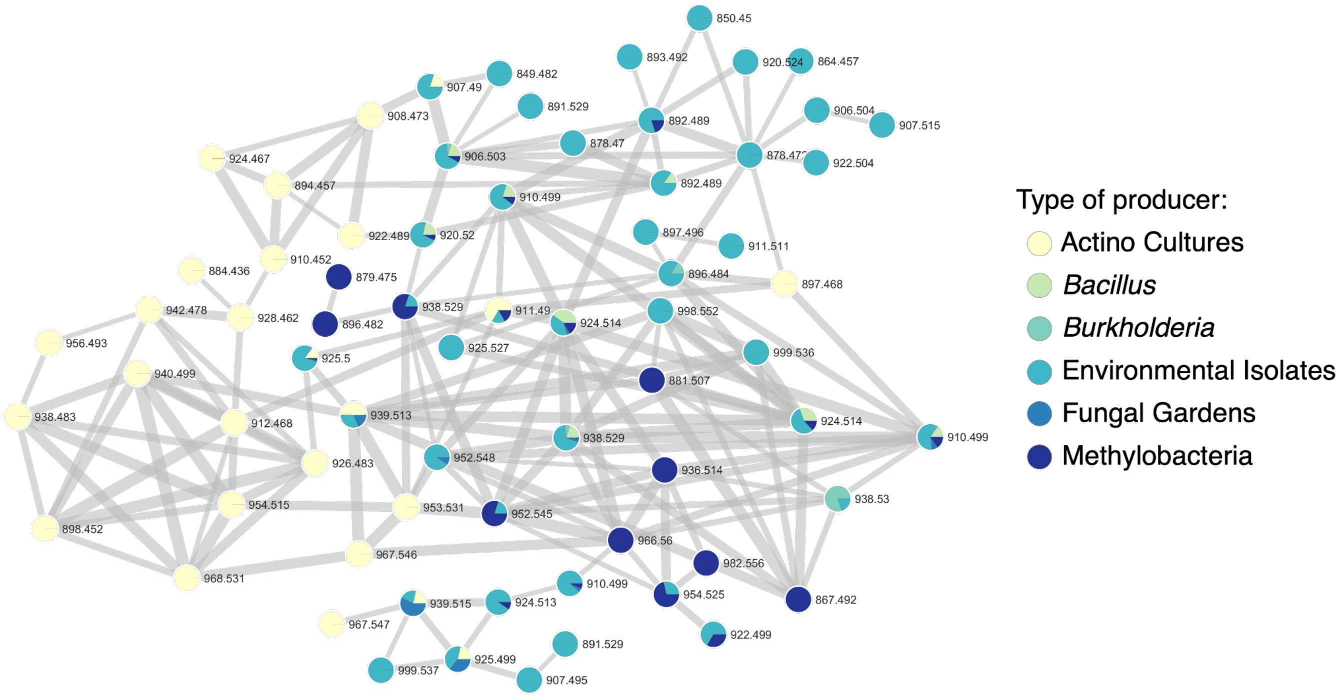

Figure S25. Detailed molecular network of FASST-2 results, showing parent masses of nodes and taxonomy of producers.

Table S5. Media Recipes

**YEME**	**Artificial Seawater (1:1 mixture of Solutions A + B)**	
4 g yeast extract	**Solution A**	**Solution B**
10 g malt extract	415.2 g NaCl	187.9 g $MgCl_2 \cdot 6H_2O$
4 g dextrose	69.5 g Na_2SO_4	22.7 g $CaCl_2 \cdot 2H_2O$
1 L seawater	11.74 g KCl	0.428 g $SrCl_2 \cdot 6H_2O$
	3.4 g $NaHCO_3$	10 L DI water
	1.7 g KBr	
	0.45 g H_3BO_3	
	0.054 g NaF	
	10 L DI water	

Table S6. Tools used by SeqCenter for genome assembly and annotation

Tool	Version	Parameters
unicycler	0.5.0	Default parameters
quast	5.2.0	Default parameters
bakta	1.8.1	Default parameters; db version 5.0
